# Synthesis of dipolar molecular rotors as linkers for metal-organic frameworks

**DOI:** 10.3762/bjoc.15.132

**Published:** 2019-06-18

**Authors:** Sebastian Hamer, Fynn Röhricht, Marius Jakoby, Ian A Howard, Xianghui Zhang, Christian Näther, Rainer Herges

**Affiliations:** 1 Otto-Diels-Institut für Organische Chemie, Kiel University, Otto-Hahn-Platz 4, D-24118 Kiel, Germany; 2 Institute of Microstructure Technology, Karlsruhe Institute of Technology, Hermann-von-Helmholtz-Platz 1, 76344 Eggenstein-Leopoldshafen, Germany; 3 Fakultät für Physik, Universität Bielefeld,Universitätsstr. 25, D-33615 Bielefeld, Germany; 4 Institut für Anorganische Chemie, Kiel University, Max-Eyth-Str. 2, D-24118 Kiel, Germany,

**Keywords:** benzothiadiazole, dipolar rotor, fluorescence, large dipole moment, metal organic framework linker

## Abstract

We report the synthesis of five dicarboxylic acid-substituted dipolar molecular rotors for the use as linker molecules in metal-organic frameworks (MOFs). The rotor molecules exhibit very low rotational barriers and decent to very high permanent, charge free dipole moments, as shown by density functional theory calculations on the isolated molecules. Four rotors are fluorescent in the visible region. The linker designs are based on push–pull-substituted phenylene cores with ethynyl spacers as rotational axes, functionalized with carboxylic acid groups for implementation in MOFs. The substituents at the phenylene core are chosen to be small to leave rotational freedom in solids with confined free volumes. The dipole moments are generated by electron-donating substituents (benzo-1,3-dioxole, benzo-1,4-dioxane, or benzo-2,1,3-thiadiazole annelation) and withdrawing substituents (difluoro, or dicyano substitution) at the opposite positions of the central phenylene core. A combination of 1,4-dioxane annelation and dicyano substitution generates a theoretically predicted, very high dipole moment of 10.1 Debye. Moreover, the molecules are sufficiently small to fit into cavities of 10 Å^3^. Hence, the dipolar rotors should be ideally suited as linkers in MOFs with potential applications as ferroelectric materials and for optical signal processing.

## Introduction

Rotors are among the fundamental functional units in engineering in our macroscopic world, as well as at the molecular level. Molecular rotors have been thoroughly investigated as basic building blocks in molecular machines and other molecular architectures [1–2]. Equally interesting is the collective behaviour of ensembles of molecular rotors in two and three dimensions, i.e., on surfaces and in materials. Different strategies have been employed to prepare ordered arrays of rotors in two dimensions, such as the inclusion of rotors in channels on the surface of particular crystals [3] on metal surfaces [4–6] or in Langmuir–Blodgett films [7]. Several strategies have also been pursued to implement molecular rotors in the solid state. Crystals of linear molecular rotors [8–13], caged rotor crystals and gyroscope like molecules [14–20] as well as organosilicates [21] and metal-organic frameworks [22–25] containing molecular rotors have been synthesized by several groups.

Molecular rotors with permanent dipole moments can be oriented by an external electric field as shown by Michl [26–27] and Price [28–29], or undergo spontaneous ordering by intermolecular dipole–dipole interactions. The ultimate goal is the fabrication of an array of dipolar rotors with a ferroelectric ground state and a Curie temperature above room temperature [30]. Such materials would have a number of exciting optical and electronic properties and applications such as signal processing and imaging. Phase transition from a stochastic to an ordered state with aligned dipoles depends on the arrangement of the rotors and the strength of their interactions. MOFs [31–32] und particular SURMOFs [33–34] are ideally suited to achieve an ordered 3D arrangement and to maximize intermolecular interactions, because the dipolar rotors are used as functional units as well as building blocks for construction of the lattice (linker). Towards this end, the dipolar rotors have to meet several preconditions: 1. the dipole moments should be strong; 2. the rotors should be small; 3. the barriers to rotation should be small (<3 kcal mol^−1^); 4. the chemistry of the dipolar rotors must be compatible with MOF growth. Preconditions 1. and 2. are somewhat contradicting each other. In the most simple treatment, the dipole moment is proportional to the point charges times their distance. However, in a real chemical situation the distance between the charges cannot be arbitrarily increased because with increasing lattice parameters controlled MOF growth becomes increasingly difficult (e.g., interpenetration). Moreover, large cavities in solids are usually difficult to keep free from solvents or other impurities that would hamper free rotation. Hence, a compromise between the size and dipole moment of the rotor/linker has to be aimed at, and care has to be taken that the rotational barriers are as low as possible.

**Figure 1:**
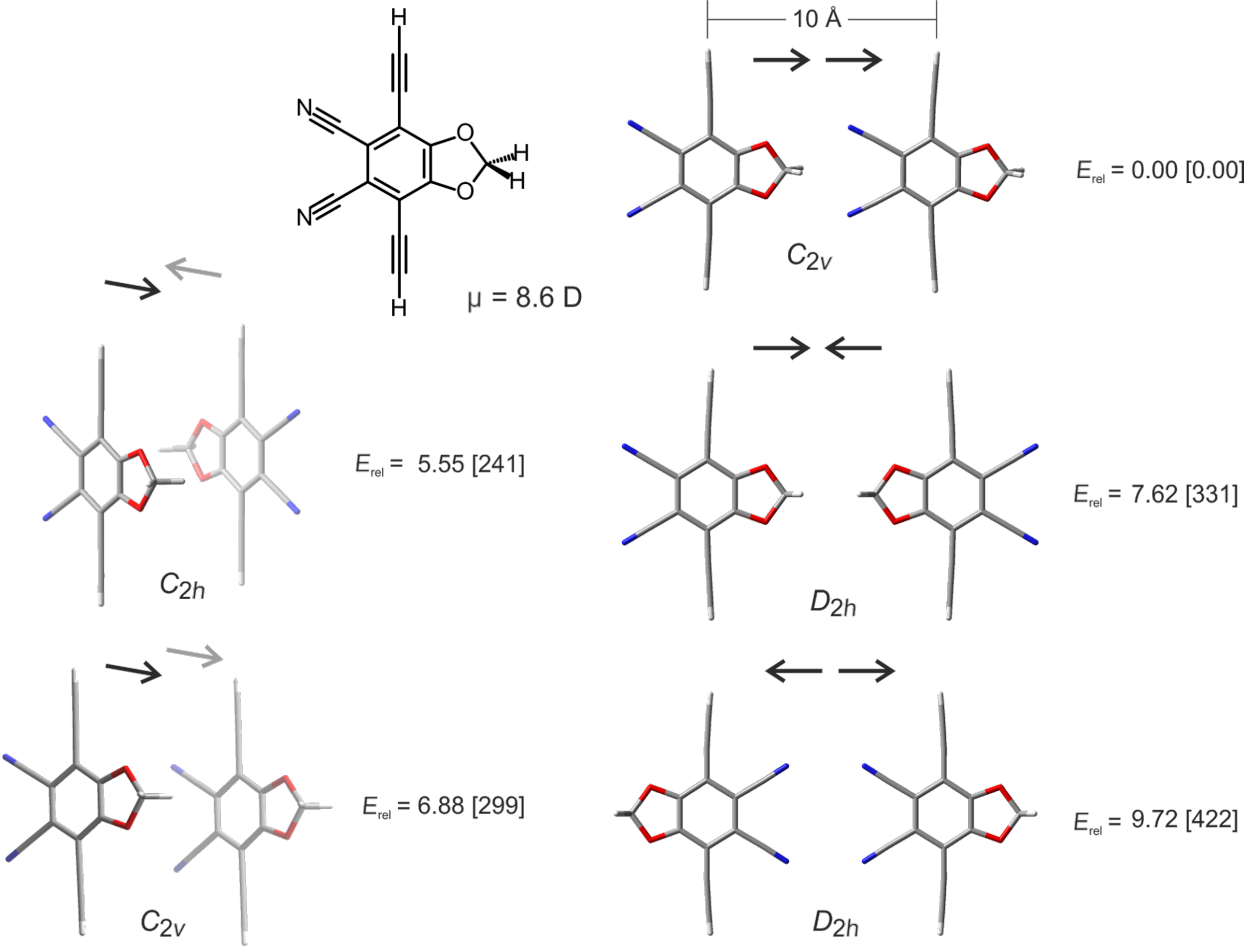
Interactions of a pair of dipolar rotors in different orientations. The axes of the rotors are parallel at a distance of 10 Å (terminal H atoms at the ethynyl units are in a plane and at an intermolecular distance of 10 Å), which is a typical distance in MOF lattices. The structures are fully optimized at the PBE-D3/defSVP level of theory within the point groups *C*_2_*_v_*, *C*_2_*_h_* and *D*_2_*_h_*. Relative energies (*E*_rel_) are given in kcal mol^−1^ and in meV (in brackets). The arrows indicate the orientation of the dipoles. The calculated dipole moment µ of the rotor (chemical structure on top left) is 8.6 D.

Figure 1 shows the interaction of a pair of dipolar rotors with parallel axes at a distance of 10 Å, which is a typical distance of the linkers in a MOF. Five different orientations in *C*_2_*_v_*, *C*_2_*_h_* and *D*_2_*_h_* symmetry are considered. The energy difference between the most favourable and the most unfavourable orientation is 9.7 kcal mol^−1^. It is important to note that the energy states of 3D ensembles of dipoles assuming periodic boundary conditions are extremely difficult to predict. Calculations of this type would be far beyond the scope of this paper. Figure 1 merely gives an approximate idea how large pairwise dipolar interactions could be. However, we dare to draw the conclusion that building a ferroelectric MOF at room temperature might not be a completely unrealistic endeavour.

**Figure 2:**
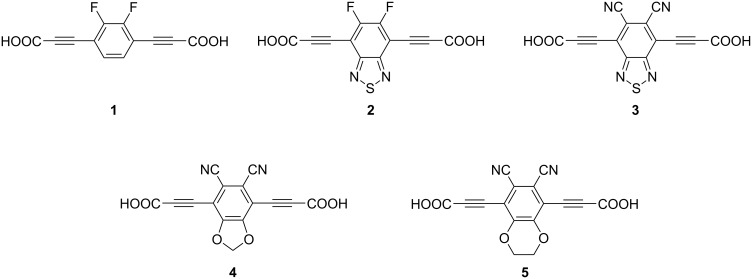
Structures of molecular dipolar rotors/linker molecules **1**–**5**.

Here we report on the synthesis of five different dipolar rotors (Figure 2) that are designed to meet the criteria 1–4 listed above, for the use as building blocks in the construction of functional MOFs.

## Results and Discussion

### Linker design and quantum chemical calculations

Aiming at high dipole moments our design was inspired by recent reports of Müllen et al. who reported on very high dipole moments of 1,2-dicyano-4,5-diamino-substituted phenyl derivatives [35]. Unfortunately, amino substituents are not compatible with MOF growth. Cyano substituents could eventually also interfere with the coordination chemistry of MOF formation. We therefore considered benzoannelation with 1,3-dioxole (**4**) and 1,4-dioxane (**5**) units as electron-donating substituents instead of amino units and substitution with fluorine (**1** and **2**) to replace the cyano units. Almost free rotation is provided by ethynyl units as rotary joints. Carboxylate groups are terminating the axes on both sides because they are known to form the typical paddle wheel structures in MOFs [36]. Benzo-annelated 2,1,3-thiadiazoles were introduced (**2** and **3**) to shift absorption and fluorescence into the visible region and to implement interesting optoelectronic properties into the final MOF structures [37–41]. Prior to synthesis, we calculated the dipole moments and barriers to rotation of the isolated rotors **1**–**5** (Table 1 and Figure 1). The rotational barriers of all rotors are below 2 kcal mol^−1^. We interpret these data as the lower limits of the rotational barriers in a MOF environment. Interactions with other linkers or neighbouring dipoles probably will increase the barriers. The dipole moments of the cyano-substituted rotors are considerably higher than those with fluoride substitution. The annelated thiadiazole ring is rather electron withdrawing and reduces the dipole moment in combination with fluoride substitution to almost zero. Compound **5** in relation to its size has an exceptionally high dipole moment of 10.1 D; thus being in the same range as 1,2-dicyano-4,5-diaminobenzene of Müllen et al. [32] without compromising MOF compatibility by 4,5-diamino substitution.

**Table 1:** DFT calculated dipole moments and rotational barriers of the dipolar rotors **1**–**5**. Geometry optimizations (symmetry **1**–**4**: *C*_2_*_v_*; **5**: *C*_2_) were performed with B3LYP/aug-cc-pVTZ level of theory and subsequent Mullikan dipole moment analysis (analogue to Müllen et al. [32]). For an appraisal of rotational barriers rotational scans were performed at the PBE/def2SVP level of density functional theory. For this evaluation both carboxylic groups where fixed in a plane. In all cases, barriers for rotations are below 2 kcal mol^−1^. In the absence of intermolecular interactions there should be thermally excited rotation at room temperature and down to very low temperatures.

Linker	µ_calc._ [D]	Δ*E*_max. calc._ [kcal mol^−1^]

**1**	2.6	1.8
**2**	0.7	1.2
**3**	6.2	0.9
**4**	8.6	1.7
**5**	10.1	1.6

### Synthesis of dipolar molecular rotor linkers

**Scheme 1:**
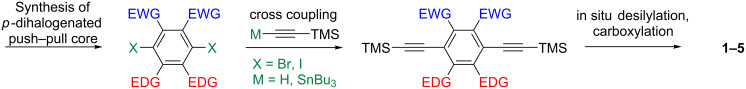
General synthetic strategy to prepare the dipolar rotors **1**–**5**.

The key step for the synthesis of all reported linkers is the coupling of a substituted aromatic core unit with two ethynyl substituents in *para*-orientation as spacer units and axis of rotation (Scheme 1). The aromatic core carries the dipole-generating substituents as well as two halogen atoms for cross coupling. For all syntheses, either terminal trimethylsilylacetylene or 1-trimethylsilyl-2-tributylstannylacetylene was employed for the coupling step followed by subsequent direct conversion of the TMS-protected acetylenes to the dicarboxylic acids using a method of Kondo and co-workers [42]. Following this procedure, purification of TMS-protected compounds was more convenient as compared to the tedious work-up with carboxylic acid derivatives directly obtained from cross coupling. Using this method, dicarboxylic acids could be obtained from simple aqueous work-up and extraction.

### Difluoro compound **1**

Difluorobenzenes have been employed as dipolar rotors by Garcia-Garibay in crystals [10], as an elongated MOF linker by Blight and Forgan [25] and for the investigation of rotor dynamics and dipole interactions in crystals by Price [28]. The predicted dipole moment of **1** with 2.6 D is small, but the molecule should be suitable for MOF preparation because the structurally similar parent compound 1,4-benzenedipropynoic acid has been successfully used for MOF synthesis [43].

**Scheme 2:**
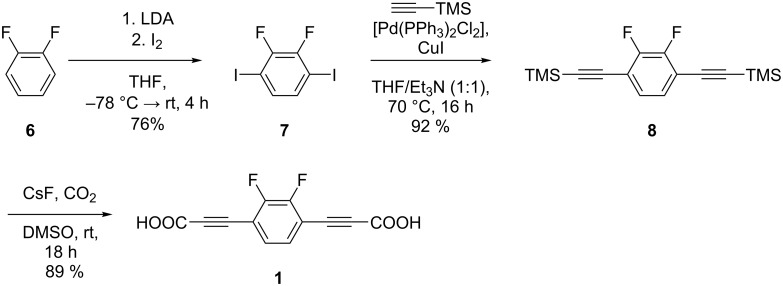
Synthesis of 3,3'-(2,3-difluoro-1,4-phenylene)dipropiolic acid (**1**) starting with diiodination of 1,2-difluorobenzene (**6**), followed by Sonogashira reaction with trimethylsilylacetylene and subsequent direct conversion to the dicarboxylic acid.

The synthesis of linker **1** was straightforward (Scheme 2). The core unit was synthesized by simple di-iodination of commercially available 1,2-difluorobenzene (**6**). The lithiation and subsequent metal iodine exchange has already been described in the literature [44–46]. While in known procedures **7** is obtained over two mono-iodination steps, we report here the di-iodination in a single step. Subsequently, **7** was reacted in a Sonogashira cross coupling with trimethylsilylacetylene to give 1,4-bis(2-trimethylsilylethynyl)-2,3-difluorobenzene (**8**). Finally, **8** was converted to the dicarboxylic acid **1** using cesium fluoride under a carbon dioxide atmosphere.

### Difluoro- and dicyanobenzothiadiazole compounds **2** and **3**

Substituted benzothiadiazole derivatives are well studied in the literature and are of great interest in organic photovoltaics and electronics. As there are no reports of such building blocks in MOF systems yet, both derivatives are promising starting materials for the preparation of functional materials based on MOF structures.

**Scheme 3:**
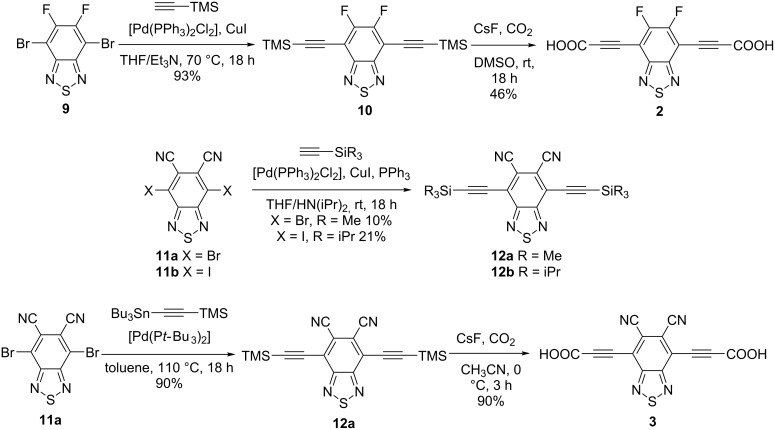
Synthesis of 3,3'-(5,6-Difluoro-2,1,3-benzothiadiazol-4,7-diyl)dipropiolic acid (**2**) and 3,3'-(5,6-Dicyano-2,1,3-benzothiadiazol-4,7-diyl)dipropiolic acid (**3**) as well as their silyl protected intermediates.

Annelated benzothiadiazole linkers could also be obtained following the general synthetic approach as shown in Scheme 3. Synthesis of the difluoro derivative **2** starts from the known compound 4,7-dibromo-5,6-difluoro-2,1,3-benzothiadiazole (**9**) which can be obtained in a four to five step reaction pathway from commercial compounds as described several times in the literature [47–50]. 5,6-Difluoro-4,7-bis(2-trimethylsilylethynyl)-2,1,3-benzothiadiazole (**10**) could be obtained in a Sonogashira reaction with trimethylsilylacetylene. Conversion to the dicarboxylic acid **2** was achieved using cesium fluoride under a carbon dioxide atmosphere.

Not quite as straightforward was the synthesis of the dicyano derivative **3**. While the synthesis of 5,6-dicyano-4,7-diiodo-2,1,3-benzothiadiazole (**11b**) was reported by Blakey, Marder and co-workers [51], no cross-coupling reactions using this derivative have been reported yet. Recently, the synthesis of 4,7-dibromo-5,6-dicyano-2,1,3-benzothiadiazole (**11a**) was also reported alongside with its use in a Sonogashira reaction [52]. The cross coupling following a Sonogashira protocol was problematic though in our case. Phthalonitrile (1,2-dicyanobenzene) units are known to form phthalocyanines and homologues, especially under harsher reaction conditions, such as the long reaction times and metal catalysis in Sonogashira reactions [53]. In the reported procedure, a bulky substituted acetylene derivative was used, probably supressing the formation of the phthalocyanine byproduct to some extent. Indeed, 5,6-dicyano-4,7-bis-(2-trimethylsilylethynyl)-2,1,3-benzothiadiazole (**12a**) could be synthesized by Sonogashira coupling in a low yield of 10% alongside with phthalocyanine byproducts. The triisopropylsilyl-protected derivative **12b** could be obtained in a slightly higher yield of 21%, presumably due to the bulkier TIPS-groups. A crystal structure of this compound was obtained (see Supporting Information File 1). To increase the yield and avoid these side reactions, we reacted diiodo compound **11b** with 1-trimethylsilyl-2-tributylstannylacetylene in a Stille cross coupling, where no formation of phthalocyanine or similar side products was observed. Changing the catalyst from tetrakis(triphenylphosphine)palladium(0) to bis(tri-*tert*-butylphosphine)palladium(0) the yield increased to satisfying 90%. For conversion into the dicarboxylic acid **3**, another change of the general procedure was necessary. Probably because of the extremely electron deficient aromatic system in **12a**, desilylation at room temperature led to polymerisation of the acetylides. No deprotected terminal acetylene was observed. To avoid polymerization, the solvent was changed to acetonitrile and the temperature was lowered to 0 °C. Additionally, carbon dioxide was not just used as the reaction atmosphere, but directly bubbled through the solution. Following this procedure, 3,3'-(5,6-dicyano-2,1,3-benzothiadiazole-4,7-diyl)dipropiolic acid (**3**) was obtained in a satisfying yield of 90%.

### Dicyanobenzodioxole and -benzodioxane compounds **4** and **5**

With the two dicyanodioxoalkylene derivatives **4** and **5**, we aimed at particularly high dipole moments. Cyano groups are known to exhibit the highest electron-withdrawing effect among the uncharged small functional groups [54]. As electron-donating substitutents, amino groups exhibit the strongest effects, especially if tris-alkyl substituted. However, we chose alkoxy substituents for our design, because phenylenediamine (1,2-diaminobenzene) units are strongly coordinating ligands which interfere with MOF formation. Moreover, alkoxy substituents provide several advantages in synthesis. While methoxy substituents are known and common donors, calculations showed that bridged alkoxy substituents such as the benzodioxole and benzodioxane groups show an even stronger electron-donating effect than two methoxy substituents while simultaneously being less sterically demanding as rotors in coordination networks. According to our calculations dioxane derivative **5** exhibits a dipole moment exceeding 10 Debye, which is, to the best of our knowledge, the highest dipole moment reported for molecules that are suitable as MOF linkers.

**Scheme 4:**
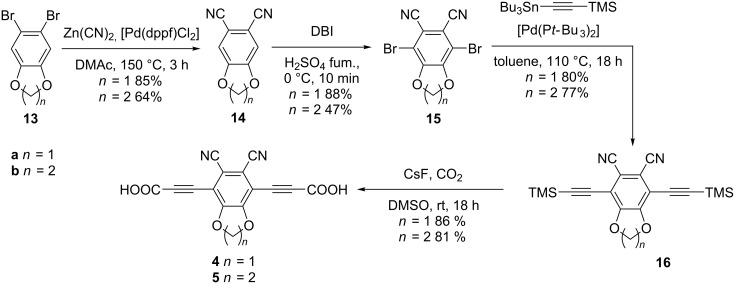
Synthesis of 3,3'-(5,6-dicyano-1,3-benzodioxole-4,7-diyl)dipropiolic acid (**4**) and 3,3'-(6,7-dicyano-1,4-benzodioxole-5,8-diyl)dipropiolic acid (**5**). DBI = Dibromoisocyanuric acid.

Both dipolar rotor units were synthesized according to a procedure outlined in Scheme 4. While the dioxane compound **5** exhibits a higher dipole moment, the dioxole compound **4** was obtained in higher yields. Starting from commercially available 5,6-dibromo-1,3-benzodioxole (**13a**) and literature known 6,7-dibromo-1,4-benzodioxane (**13b**) [55], dicyanation of the dibromo compounds was achieved with zinc(II) cyanide under palladium catalysis [56]. The typical Rosemund–von-Braun reaction using copper(I) cyanide was reported before for the preparation of benzodioxole derivative **13a** [57–58], but gave far inferior yields and was hampered by a tedious work-up. The dicyano compounds 5,6-dicyano-1,3-benzodioxole (**14a**) and 6,7-dicyano-1,4-benzodioxane (**14b**) were dibrominated after a protocol using dibromoisocyanuric acid in fuming sulfuric acid [59]. Subsequent Stille cross coupling (to avoid phthalocyanine formation) using conditions established in the synthesis of linker **3** gave the bis(trimethylsilylethynyl) compounds **15a** and **15b** in nearly quantitative yields. Analogously to the above-described systems, conversion into the respective dicarboxylic acids **4** and **5** took place by reaction with cesium fluoride under a carbon dioxide atmosphere.

## Conclusion

In summary, we reported here the synthesis of five dipolar rotors consisting of a push–pull-substituted phenylene core, with two ethynyl units in *para-*position as the rotary axis and two dicarboxylic acids for the use as MOF linkers. Linkers **2** and **3** contain substituted 2,1,3-benzothiadiazole rotor units, with absorption and emission wavelengths in the visible region (see Supporting Information File 2). The dipolar rotors **4** and **5** exhibit the largest dipole moments known for MOF linkers so far, with **5** even exceeding 10 Debye. Cross-coupling reactions, particularly Sonogashira reactions of dicyanobenzenes are known to be accompanied by phthalocyanine formation and other side products. These problems are avoided by applying Stille conditions using 1-trimethylsilyl-2-tributylstannylacetylene in combination with the bulky palladium catalyst bis(tri-*tert*-butylphosphino)palladium. The TMS-protected, dicyano-substituted rotors with thiadiazole, dioxole and dioxane annelation **12a**, and **16** were prepared in yields exceeding 90%. Thus, the dipolar rotors **1**–**5** are now available in gram amounts for the synthesis of MOF or SURMOF structures with promising electric and optical properties.

## Supporting Information

File 1 Experimental procedures, ^1^H and ^13^C NMR spectra of new compounds as well as UV–vis and fluorescence spectra of compounds **1**–**5** and crystallographic data for compound **12b**.

File 2 cif file for compound **12b**.

File 3 checkcif report for compound **12b**.

## References

[1] Balzani V, Credi A, Raymo F M, Stoddart J F (2000). Angew Chem, Int Ed.

[2] Browne W R, Feringa B L (2006). Nat Nanotechnol.

[3] Dron P I, Zhao K, Kaleta J, Shen Y, Wen J, Shoemaker R K, Rogers C T, Michl J (2016). Adv Funct Mater.

[4] Baber A E, Tierney H L, Sykes E C H (2008). ACS Nano.

[5] Otte F L, Lemke S, Schütt C, Krekiehn N R, Jung U, Magnussen O M, Herges R (2014). J Am Chem Soc.

[6] Baisch B, Raffa D, Jung U, Magnussen O M, Nicolas C, Lacour J, Kubitschke J, Herges R (2009). J Am Chem Soc.

[7] Kaleta J, Wen J, Magnera T F, Dron P I, Zhu C, Michl J (2018). Proc Natl Acad Sci U S A.

[8] Dominguez Z, Khuong T-A V, Dang H, Sanrame C N, Nuñez J E, Garcia-Garibay M A (2003). J Am Chem Soc.

[9] Rodríguez-Molina B, Ochoa M E, Romero M, Khan S I, Farfán N, Santillan R, Garcia-Garibay M A (2013). Cryst Growth Des.

[10] Arcos-Ramos R, Rodriguez-Molina B, Gonzalez-Rodriguez E, Ramirez-Montes P I, Ochoa M E, Santillan R, Farfán N, Garcia-Garibay M A (2015). RSC Adv.

[11] Catalano L, Pérez-Estrada S, Terraneo G, Pilati T, Resnati G, Metrangolo P, Garcia-Garibay M A (2015). J Am Chem Soc.

[12] Pérez-Estrada S, Rodríguez-Molina B, Xiao L, Santillan R, Jiménez-Osés G, Houk K N, Garcia-Garibay M A (2015). J Am Chem Soc.

[13] Jiang X, O’Brien Z J, Yang S, Lai L H, Buenaflor J, Tan C, Khan S, Houk K N, Garcia-Garibay M A (2016). J Am Chem Soc.

[14] Zhang X, Shao X-D, Li S-C, Cai Y, Yao Y-F, Xiong R-G, Zhang W (2015). Chem Commun.

[15] Setaka W, Yamaguchi K (2013). J Am Chem Soc.

[16] Setaka W, Inoue K, Higa S, Yoshigai S, Kono H, Yamaguchi K (2014). J Org Chem.

[17] Masuda T, Arase J, Inagaki Y, Kawahata M, Yamaguchi K, Ohhara T, Nakao A, Momma H, Kwon E, Setaka W (2016). Cryst Growth Des.

[18] Fujiwara A, Inagaki Y, Momma H, Kwon E, Yamaguchi K, Kanno M, Kono H, Setaka W (2017). CrystEngComm.

[19] Tsurunaga M, Inagaki Y, Momma H, Kwon E, Yamaguchi K, Yoza K, Setaka W (2018). Org Lett.

[20] Nawara A J, Shima T, Hampel F, Gladysz J A (2006). J Am Chem Soc.

[21] Bracco S, Beretta M, Cattaneo A, Comotti A, Falqui A, Zhao K, Rogers C, Sozzani P (2015). Angew Chem, Int Ed.

[22] Winston E B, Lowell P J, Vacek J, Chocholoušová J, Michl J, Price J C (2008). Phys Chem Chem Phys.

[23] Jiang X, Duan H-B, Khan S I, Garcia-Garibay M A (2016). ACS Cent Sci.

[24] Vogelsberg C S, Uribe-Romo F J, Lipton A S, Yang S, Houk K N, Brown S, Garcia-Garibay M A (2017). Proc Natl Acad Sci U S A.

[25] Marshall R J, Kalinovskyy Y, Griffin S L, Wilson C, Blight B A, Forgan R S (2017). J Am Chem Soc.

[26] Vacek J, Michl J (2001). Proc Natl Acad Sci U S A.

[27] Horinek D, Michl J (2003). J Am Chem Soc.

[28] Horansky R D, Clarke L I, Price J C, Khuong T-A V, Jarowski P D, Garcia-Garibay M A (2005). Phys Rev B.

[29] Horansky R D, Clarke L I, Winston E B, Price J C, Karlen S D, Jarowski P D, Santillan R, Garcia-Garibay M A (2006). Phys Rev B.

[30] Michl J, Sykes E C H (2009). ACS Nano.

[31] Furukawa H, Cordova K E, O'Keeffe M, Yaghi O M (2013). Science.

[32] Zhou H-C, Kitagawa S (2014). Chem Soc Rev.

[33] Liu J, Wöll C (2017). Chem Soc Rev.

[34] Heinke L, Cakici M, Dommaschk M, Grosjean S, Herges R, Bräse S, Wöll C (2014). ACS Nano.

[35] Wudarczyk J, Papamokos G, Margaritis V, Schollmeyer D, Hinkel F, Baumgarten M, Floudas G, Müllen K (2016). Angew Chem, Int Ed.

[36] Farha O K, Hupp J T (2010). Acc Chem Res.

[37] Parker T C, Patel D G, Moudgil K, Barlow S, Risko C, Brédas J-L, Reynolds J R, Marder S R (2015). Mater Horiz.

[38] Blouin N, Michaud A, Gendron D, Wakim S, Blair E, Neagu-Plesu R, Belletête M, Durocher G, Tao Y, Leclerc M (2008). J Am Chem Soc.

[39] Zhou H, Yang L, Price S C, Knight K J, You W (2010). Angew Chem, Int Ed.

[40] Casey A, Dimitrov S D, Shakya-Tuladhar P, Fei Z, Nguyen M, Han Y, Anthopoulos T D, Durrant J R, Heeney M (2016). Chem Mater.

[41] Nielsen C B, White A J P, McCulloch I (2015). J Org Chem.

[42] Yonemoto-Kobayashi M, Inamoto K, Tanaka Y, Kondo Y (2013). Org Biomol Chem.

[43] Gomez-Gualdron D A, Gutov O V, Krungleviciute V, Borah B, Mondloch J E, Hupp J T, Yildirim T, Farha O K, Snurr R Q (2014). Chem Mater.

[44] Rausis T, Schlosser M (2002). Eur J Org Chem.

[45] Ramirez-Montes P I, Ochoa M E, Santillan R, Ramírez D J, Farfán N (2014). Cryst Growth Des.

[46] Tsukada S, Kondo M, Sato H, Gunji T (2016). Polyhedron.

[47] Zhang Y, Chien S-C, Chen K-S, Yip H-L, Sun Y, Davies J A, Chen F-C, Jen A K-Y (2011). Chem Commun.

[48] Chen Z, Cai P, Chen J, Liu X, Zhang L, Lan L, Peng J, Ma Y, Cao Y (2014). Adv Mater (Weinheim, Ger).

[49] Jeong I, Chae S, Yi A, Kim J, Chun H H, Cho J H, Kim H J, Suh H (2017). Polymer.

[50] Viswanathan V N, Rao A D, Pandey U K, Kesavan A V, Ramamurthy P C (2017). Beilstein J Org Chem.

[51] Shi Q, Zhang S, Zhang J, Oswald V F, Amassian A, Marder S R, Blakey S B (2016). J Am Chem Soc.

[52] Wudarczyk J, Papamokos G, Marszalek T, Nevolianis T, Schollmeyer D, Pisula W, Floudas G, Baumgarten M, Müllen K (2017). ACS Appl Mater Interfaces.

[53] Kopylovich M N, Kukushkin V Y, Haukka M, Luzyanin K V, Pombeiro A J L (2004). J Am Chem Soc.

[54] Cheng L T, Tam W, Stevenson S H, Meredith G R, Rikken G, Marder S R (1991). J Phys Chem.

[55] Hellberg J, Dahlstedt E, Pelcman M E (2004). Tetrahedron.

[56] Iqbal Z, Lyubimtsev A, Hanack M (2008). Synlett.

[57] Metz J, Schneider O, Hanack M (1984). Inorg Chem.

[58] Lawrence D S, Whitten D G (1996). Photochem Photobiol.

[59] Wang J, Khanamiryan A K, Leznoff C C (2004). J Porphyrins Phthalocyanines.

