# Supplementary material for: Synthesis of dipolar molecular rotors as linkers for metal-organic frameworks
Source: Beilstein J Org Chem. 2019 Jun 18;15:1331–8. doi: 10.3762/bjoc.15.132 (PMC6604738; doi:10.3762/bjoc.15.132)
Supplement: File 1 — Experimental procedures, 1H and 13C NMR spectra of new compounds as well as UV–vis and fluorescence spectra of compounds 1–5 and crystallographic data for compound 12b. [file Beilstein_J_Org_Chem-15-1331-s001.pdf]

BEILSTEIN JOURNAL OF ORGANIC CHEMISTRY

Supporting Information

for

Synthesis of dipolar molecular rotors as linkers for metal-organic frameworks

Sebastian Hamer, Fynn Röhricht, Marius Jakoby, Ian A. Howard, Xianghui Zhang, Christian Näther and Rainer Herges

Beilstein J. Org. Chem. **2019,** *15,* 1331–1338. doi:10.3762/bjoc.15.132

Experimental procedures, ^{1}H and ^{13}C NMR spectra of new compounds as well as UV–vis and fluorescence spectra of compounds 1–5 and crystallographic data for compound 12b

License and Terms: This is a supporting information file under the terms of the Creative Commons Attribution License (http://creativecommons.org/licenses/by/4.0). Please note that the reuse, redistribution and reproduction in particular requires that the authors and source are credited. The license is subject to the *Beilstein Journal of Organic Chemistry* terms and conditions: (https://www.beilstein-journals.org/bjoc)

Analytical equipment, experimental procedures, spectral data and crystallographic data

Table of contents

I.	General methods and analytical equipment	S3
II.	Experimental procedures and NMR spectra	S5
III.	UV–vis and fluorescence spectra	S36
IV.	Crystallographic data for compound **12b**	S37

I. General methods and analytical equipment

Materials

If not otherwise noted, all reagents were purchased from commercial suppliers and were used as received. 5,6-difluoro-4,7-dibromo-2,1,3-benzothiadiazole (**9**),[1, 2, 3, 4] 4,7-dibromo-5,6-dicyano-2,1,3-benzothiadiazole (**11a**),[5] 5,6-dicyano-4,7-diiodo-2,1,3-benzothiadiazole (**11b**),[6] 6,7-dibromo-1,4-benzodioxane (**13b**)[7] and 1-trimethylsilyl-2-tributylstannylacetylene[8] were prepared according to literature procedures.

NMR spectroscopy

NMR spectra were measured in deuterated solvents (Deutero). All spectra were referenced to the corresponding solvent residual peak [9]:

**solvent**	**degree of deuteration**	**^{1}H signal**	**^{13}C signal**
**chloroform-*d*$_1$**	99.8 %	7.26 (s)	77.16 (t)
**acetone-*d*$_6$**	99.8 %	2.05 (quin)	29.84 (sept)
**DMSO-*d*$_6$**	99.8 %	2.50 (s)	39.52 (sept)

NMR measurements were performed with a Bruker DRX 500 (^{1}H NMR: 500 MHz, ^{13}C NMR: 125 MHz, ^{19}F NMR: 470 MHz) and a Bruker AV 600 (^{1}H NMR: 600 MHz, ^{13}C NMR: 150 MHz) spectrometer. Signals were abbreviated with s, s_b, d, t, m and m_c for singlet, broad singlet, doublet, triplet, multiplet and centered multiplet.

Melting points

Melting points were measured with a Melting Point B-560 (Büchi) in melting point tubes and were taken over without correction.

Mass spectrometry

The high resolution (HR-EI) mass spectra were measured with an AccuTOF GCv 4G (Joel) with ionization energy of 70 eV.

Infrared spectroscopy

Infrared spectra were measured on a Perkin-Elmer 1600 Series FT-IR spectrometer with an A531-G Golden-Gate-Diamond-ATR-unit. Signals were abbreviated with w, m, s, vs for weak, medium, strong and very strong signal intensity and index b for broad signals.

UV–vis and photoluminescence spectroscopy

UV–vis Absorption measurements were performed with a spectrophotometer (Perkin-Elmer Lambda 960). PL measurements were performed with a luminescence spectrometer (Cary Eclipse), with the excitation wavelength chosen to maximize the samples absorption.

Chromatography stationary phases

For column chromatography purifications silica gel (Merck, particle size 0.040–0.063 mm) was used. Flash column chromatography purfications were performed on a Biotage® type Isolera one with Biotage® Ultra cartridges (Biotage®, HP-SphereTM, particle diameter: 25 µm, cartridges sizes: 10 g, 25 g, 50 g and 100 g) were used. R_f values were determined by thin layer chromatography on Polygram® SilG/UV254 TLC plates (Macherey Nagel, 0.2 mm particle size).

II. Experimental procedures and NMR spectra

General remark on the characterization of dicarboxylic acids 1–5

The dicarboxylic acid compounds **1**–**5** were all characterized by ^{1}H and ^{13}C NMR, IR and UV–vis as well as fluorescence spectroscopy. However, EI, ESI and MALDI mass spectrometric analysis won't show peaks for intact compounds due to the lability of the carboxylic acid groups. Thus, only the decarboxylation fragments of compounds could be found. This being stated, characterization is still confident as compounds used for dicarboxylic acid synthesis are fully characterized, ^{13}C NMR spectra of **1**–**5** show the respective carboxylic acid carbon signals, ^{1}H NMR spectra don't show terminal acetylene signals, IR spectra show carboxylic acid functions and mass spectra show the decarboxylated fragments.

Synthesis of 3,3'-(2,3-difluoro-1,4-phenylene)dipropiolic acid (1)

2,3-Difluoro-1,4-diiodobenzene (7)

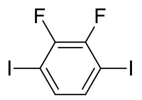

7

Under nitrogen atmosphere, a 2.5 M solution of *n*-butyllithium in *n*-hexane (24.0 mL, 60.0 mmol) was added to a solution of diisopropylamine (8.62 mL, 61.0 mmol) in anhydrous THF (100 mL) at −78 °C and stirred for 30 min. 1,2-Difluorobenzene (**6**, 2.40 mL, 24.4 mmol) was then added at −78 °C and the reaction mixture was stirred for another hour. Subsequently, an ice-cooled solution of iodine (12.4 g, 48.7 mmol) in anhydrous THF (50 mL) was added, the cooling removed and the reaction mixture stirred for further 4 hours while warming up to room temperature. A saturated solution of sodium thiosulfate (100 mL) was added, the mixture was extracted with diethylether (2 × 100 mL) and the combined organic layers were washed with saturated sodium thiosulfate solution (2 × 100 mL). The organic layer was dried over magnesium sulfate, the solvent evaporated in vacuo and the crude product purified by

column chromatography using silica and *n*-pentane as eluent (R_f = 0.45) to obtain a colourless solid (6.80 g, 18.6 mmol, 76 %).

^{1}**H NMR** (500MHz, DMSO-d6, 300 K): δ = 7.46-7.44 (m, 2H) ppm;

^{13}**C NMR** (125 MHz, DMSO-d6, 300 K): δ = 149.3 (dd, $^1J_{C,F}$ = 249.1 Hz, $^2J_{C,F}$ = 18.6 Hz, 2C), 135.5 (m_c, 2C), 84.1 (m_c, 2C) ppm;

MS (EI, 70 eV): m/z (%) = 365 (100) $[M]^+$, 238 (20) $[M\text{-}I]^+$, 112 (41) $[M\text{-}2I]^+$.

1,4-Bis-(2-trimethylsilylethinyl)-2,3-difluorobenzene (8)

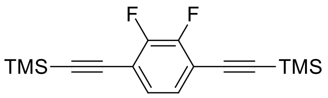

8

Under nitrogen atmosphere, 2,3-difluoro-1,4-diiodobenzene (**7**, 430 mg, 1.18 mmol), bis-(triphenylphospino)dichloropalladium(II) (85.1 mg, 121 µmol) and copper(I) iodide (23.2 mg, 122 µmol) were dissolved in anhydrous THF (20 mL) and triethylamine (20 mL). Trimethylsilylacetylene (0.41 mL, 2.96 mmol) was added and the solution stirred at 70 °C for 16 h. Subsequently, the reaction mixture was filtered through a bed of Celite, the solvent was evaporated in vacuo and the crude product purified by column chromatography using silica and *n*-hexane as eluent (R_f = 0.44) to obtain a colourless solid (332 mg, 1.08 mmol, 92%).

Melting point: 78 °C;

^{1}**H NMR** (500MHz, CDCl3, 300 K): δ = 7.12-7.10 (m, 2H) ppm;

^{13}**C NMR** (125 MHz, CDCl3, 300 K): δ = 151.4 (dd, $^1J_{C,F}$ = 255.9 Hz, $^2J_{C,F}$ = 15.0 Hz, 2C), 127.9 (m_c, 2C), 114.2 (m_c, 2C), 103.7 (m_c, 2C), 96.4 (m_c, 2C), 0.1 (6C) ppm.

IR (ATR): ῦ = 2960 (w), 2900 (w), 2165 (m), 1549 (w), 1489 (m), 1466 (s), 1310 (w), 1249 (s), 1228 (m), 1170 (w), 1044 (m), 963 (s), 838 (vs), 817 (vs), 758 (vs), 702 (s) cm^{-1};

MS (EI, 70 eV): m/z (%) = 306 (45) $[M]^+$, 293 (100) $[M\text{-}Me]^+$, 112 (41) $[M\text{-}2I]^+$;

HR-MS (EI, 70 eV): m/z = calcd. for $C_{16}H_{20}F_2Si_2$ $[M]^+$ 306.1072, found 306.1072.

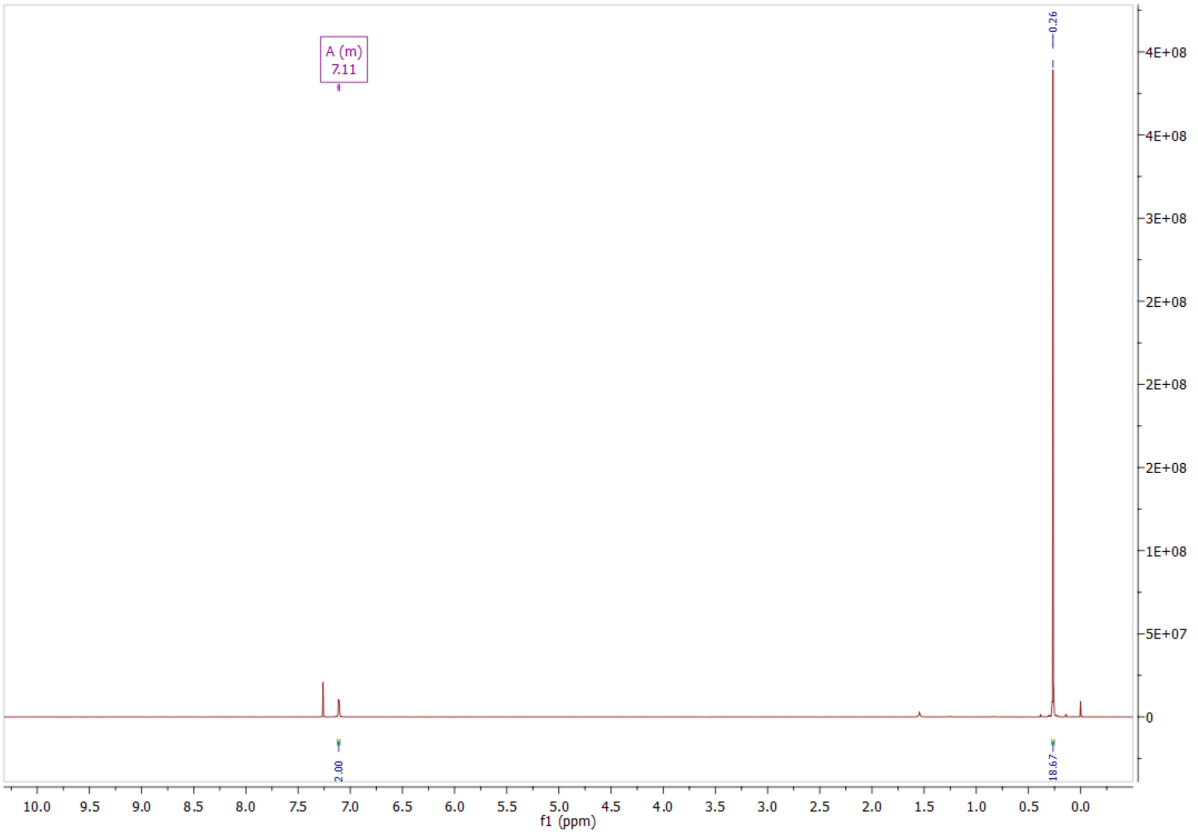

Figure S1: ^{1}H NMR spectrum of compound **8** measured in deuterated chloroform.

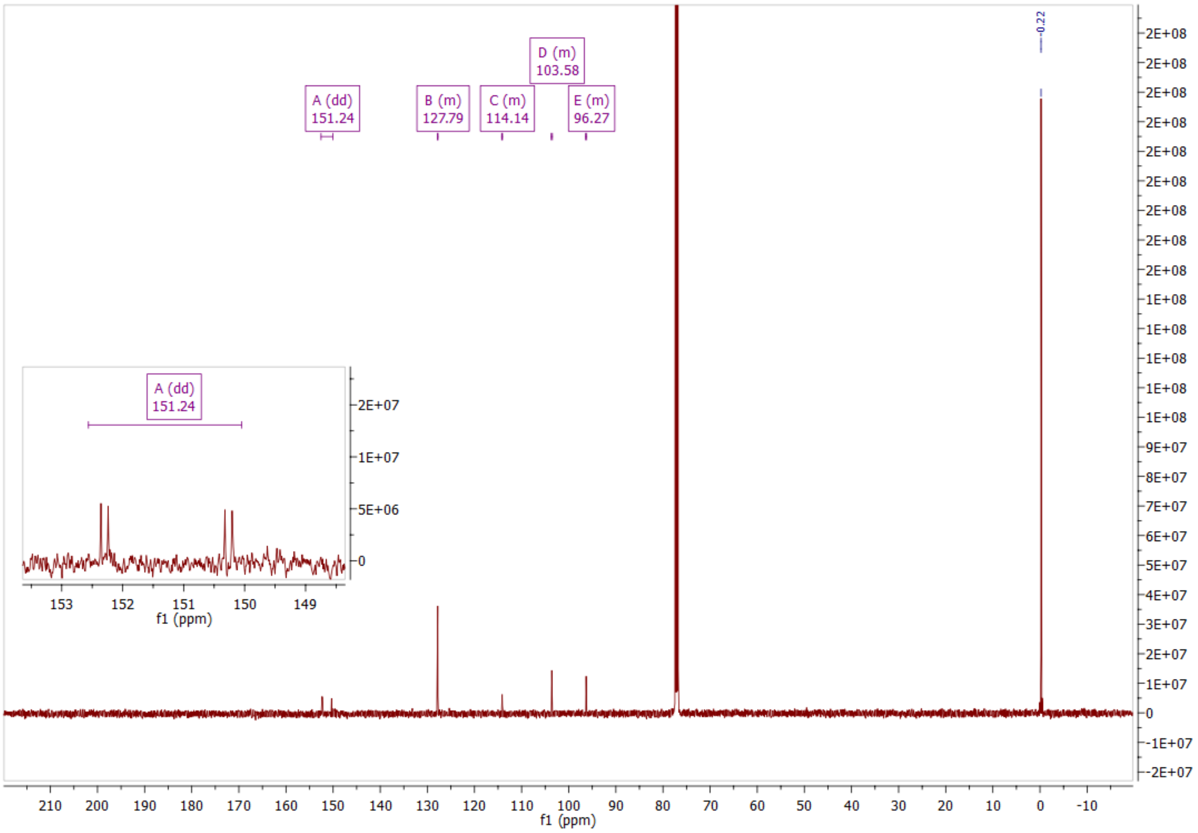

Figure S2: ^{13}C NMR spectrum of compound **8** measured in deuterated chloroform.

3,3'-(2,3-Difluoro-1,4-phenylene)dipropiolic acid (1)

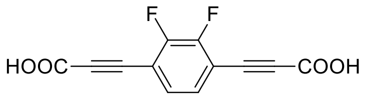

1

Under carbon dioxide atmosphere, 1,4-bis(2-trimethylsilylethinyl)-2,3-difluorobenzene (1.50 g, 4.89 mmol) was dissolved in anhydrous DMSO (10 mL), cesium fluoride (1.86 g, 12.2 mmol) was added and the mixture stirred vigorously at room temperature for 18 h. Subsequently, the reaction mixture was diluted with water (400 mL), extracted with dichloromethane (3 × 150 mL) and the aqueous layer acidified with 6 M hydrogen chloride solution. The aqueous layer was extracted with diethyl ether (4 × 50 mL), the organic layer washed with 2 M hydrogen chloride solution (200 mL), dried over magnesium sulfate and the solvents removed in vacuo to obtain a pale yellow solid (1.09 g, 4.37 mmol, 89%).

Melting point: >200 °C (slowly decomposing);

^{1}H NMR (600MHz, Acetone-d6, 300 K): δ = 7.62 (s, 2H) ppm;

^{13}C NMR (125 MHz, Aceton-d6, 300 K): δ = 153.7 (2C), 152.4 (dd, $^{1}J_{C,F}$ = 257.5 Hz, $^{2}J_{C,F}$ = 14.9 Hz, 2C), 130.3 (m_c, 2C), 113.8 (m_c, 2C), 89.1 (m_c, 2C), 76.5 (m_c, 2C) ppm;

IR (ATR): ῦ = 2843 (m_{br}), 2578 (m_{br}), 2227 (s), 1697 (s), 1597 (s), 1497 (s), 1475 (s), 1369 (s), 1287 (s), 1247 (vs), 1227 (vs), 1177 (s), 1066 (s), 1005 (s), 956 (m), 864 (s), 827 (vs), 739 (s), 694 (vs) cm^{-1};

MS (EI, 70 eV): m/z (%) = 206 (12) $[M-CO_2]^+$, 162 (100) $[M-2CO_2]^+$, 112 (9) $[C_6H_2F_2]^+$.

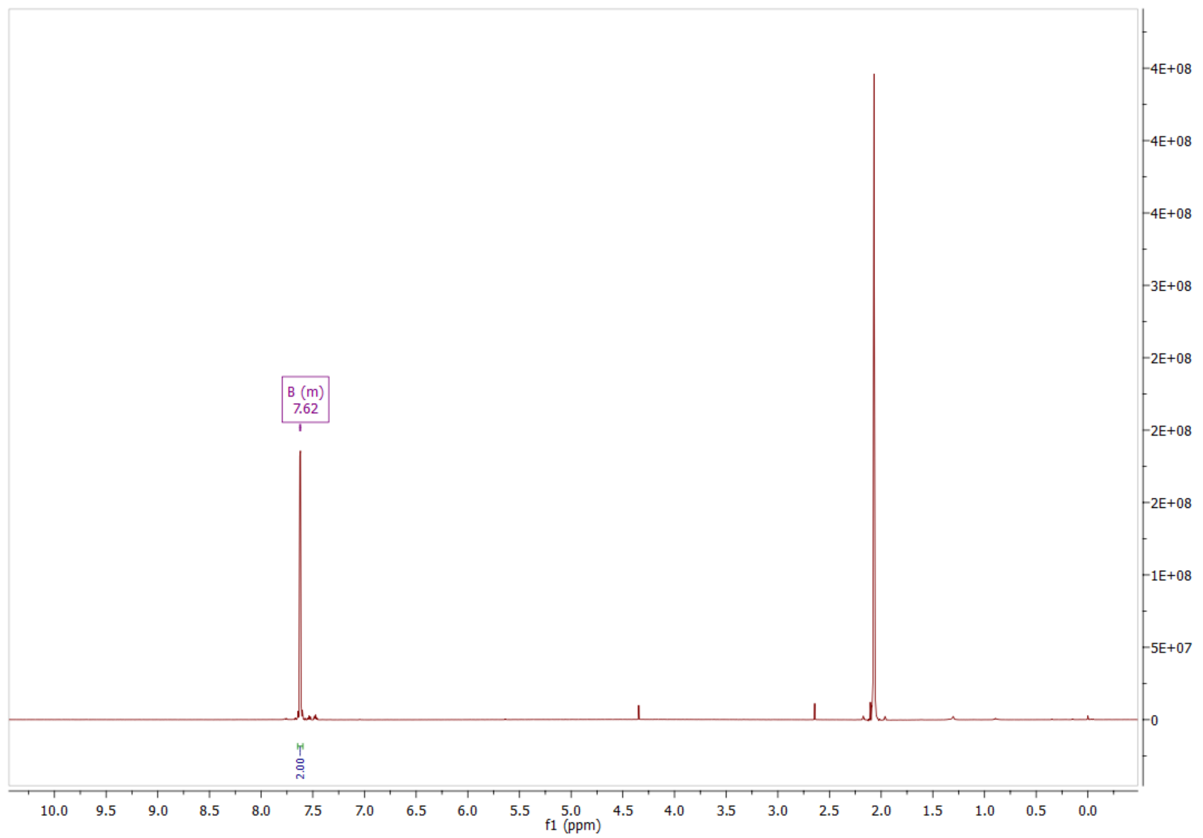

Figure S3: ^{1}H NMR spectrum of compound **1** measured in deuterated acetone.

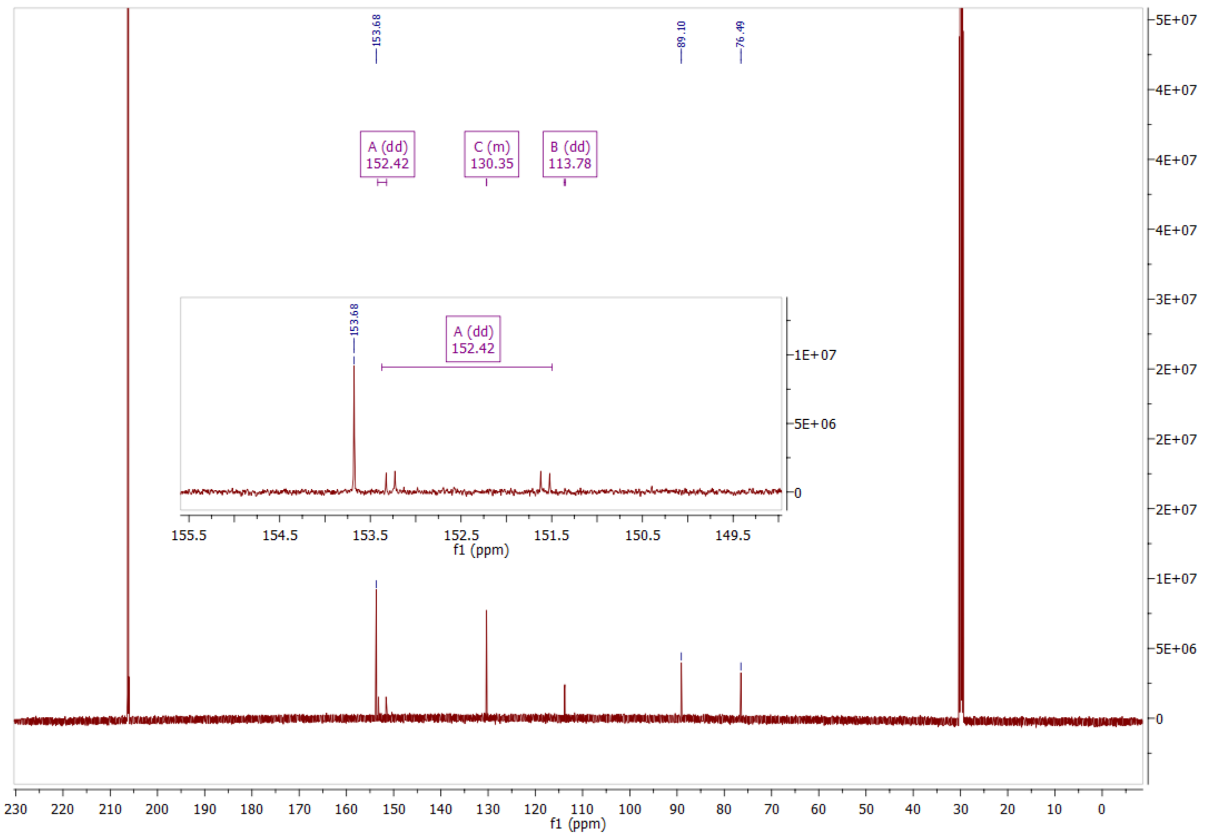

Figure S4: ^{13}C NMR spectrum of compound **1** measured in deuterated acetone.

Synthesis of 3,3'-(5,6-difluoro-2,1,3-benzothiadiazole-4,7-diyl)dipropiolic acid (2)

4,7-Bis-(2-trimethylsilylethinyl)-5,6-difluoro-2,1,3-benzothiadiazole (10)

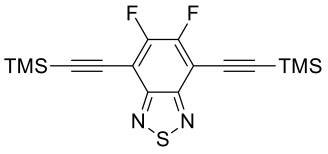

10

Under nitrogen atmosphere, 5,6-difluoro-4,7-dibromo-2,1,3-benzothiadiazole (**9**, 1.00 g, 3.03 mmol), bis-(triphenylphosphino)dichloropalladium(II) (214 mg, 305 µmol) and copper(I) iodide (59.0 mg, 310 µmol) were dissolved in abs. THF (35 mL) and triethylamine (35 mL) was added. Subsequently, trimethylsilylacetylene (1.70 mL, 11.9 mmol) was added and the solution was stirred for 18 h at 70 °C. The reaction mixture was filtered through a bed of celite, washed with dichloromethane and the solvents evaporated in vacuo. The obtained crude product was purified by column chromatography using silica and dichloromethane/*n*-pentane (1:5, R_f = 0.25) as eluent to obtain a yellow solid (1.03 g, 2.83 mmol, 93 %).

Melting point: 140 °C;

^{1}H NMR (500MHz, CDCl3, 300 K): δ = 0.34 (s, 18H) ppm;

^{13}C NMR (125 MHz, CDCl3, 300 K): δ = 155.3 (dd, $^1J_{C,F}$ = 264.7 Hz, $^2J_{C,F}$ = 19.5 Hz, 2C), 150.3 (m_c, 2C), 111.2 (m_c, 2C), 104.3 (m_c, 2C), 92.2 (m_c, 2C), -0.2 (6C) ppm;

IR (ATR): ῦ = 2960 (w), 2898 (w), 2167 (w), 1586 (w), 1544 (w), 1494 (w), 1435 (s), 1361 (m), 1345 (s), 1301 (m), 1250 (s), 1186 (w), 1145 (w), 1007 (s), 991 (s), 837 (vs), 758 (vs), 785 (s), 758 (vs), 733 (s), 701 (s), 676 (s), 659 (m) cm^{-1};

MS (EI, 70 eV): m/z (%) = 364 (44) $[M]^+$, 349 (100) $[M-Me]^+$;

HR-MS (EI, 70 eV): m/z = calcd. for $C_{16}H_{18}F_2N_2SSi_2$ $[M]^+$ 364.0697, found 364.0697.

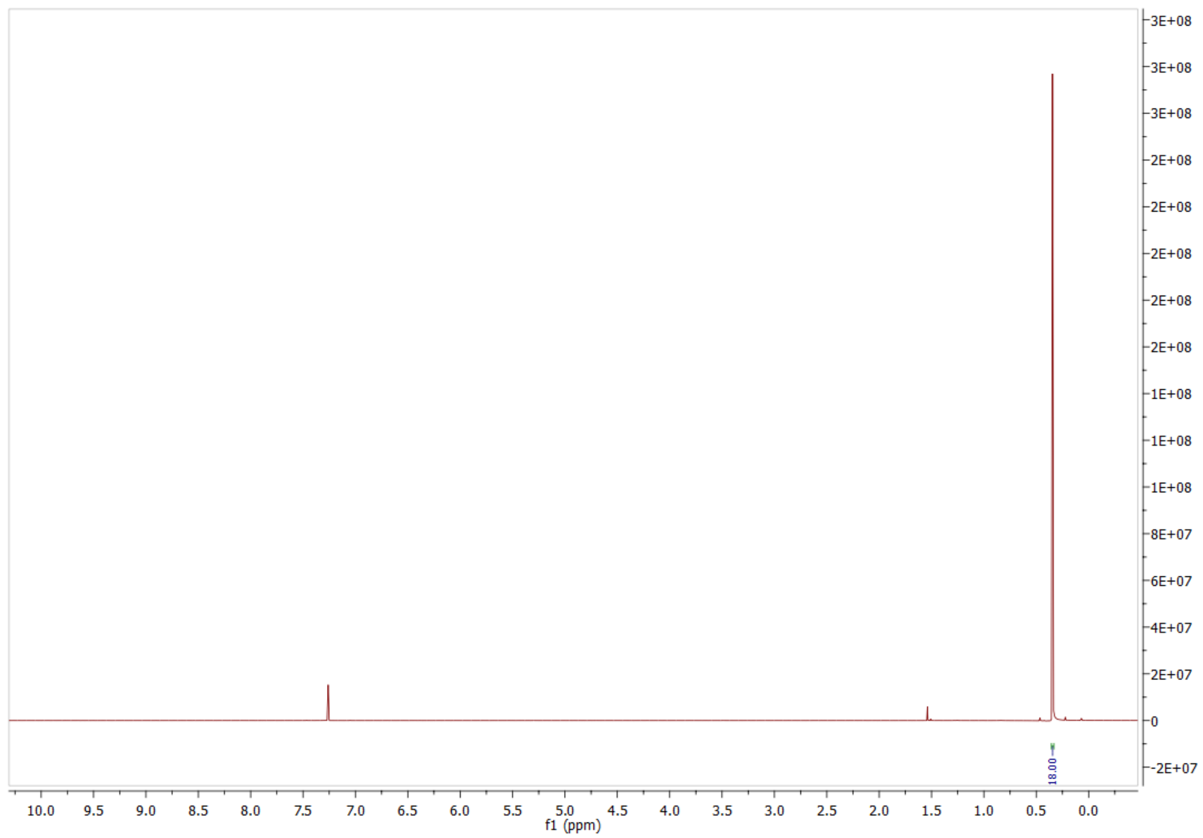

Figure S5: ^{1}H NMR spectrum of compound **10** measured in deuterated chloroform.

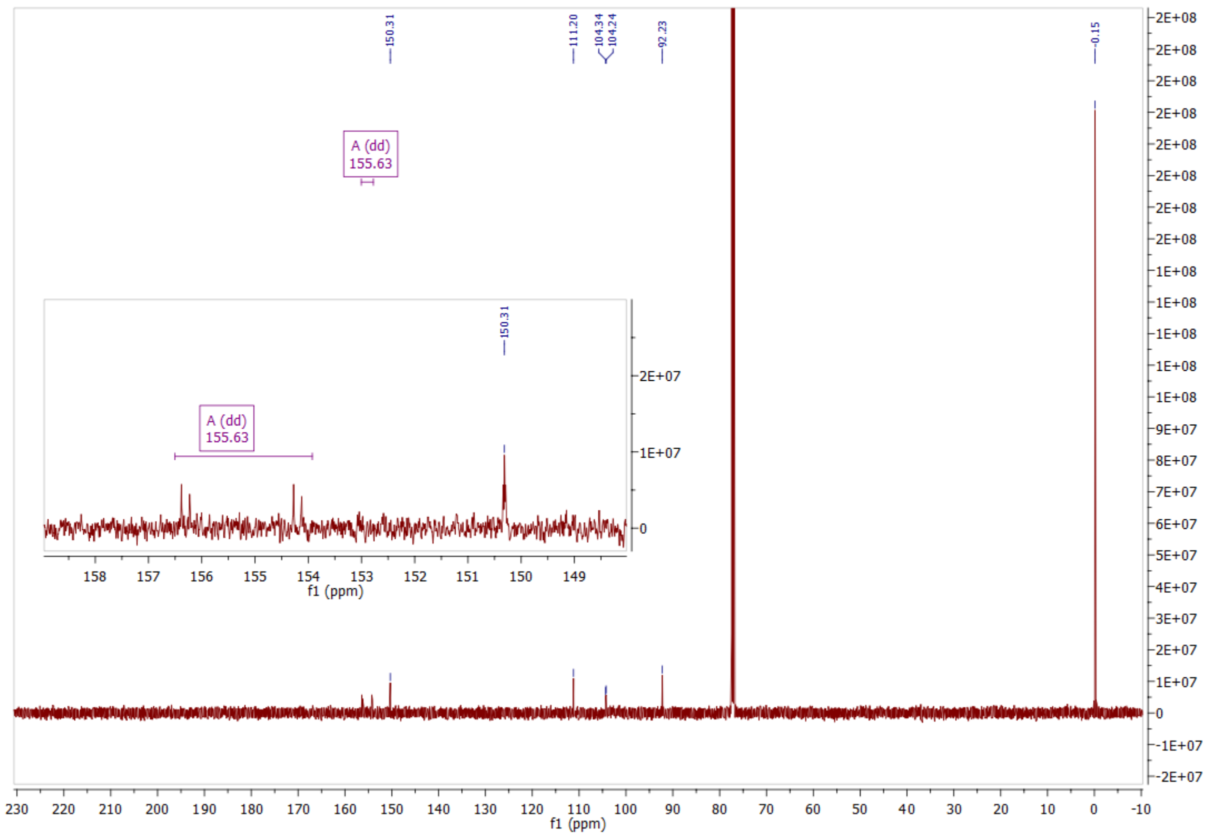

Figure S6: ^{13}C NMR spectrum of compound **10** measured in deuterated chloroform.

3,3'-(5,6-Difluoro-2,1,3-benzothiadiazole-4,7-diyl)dipropiolic acid (2)

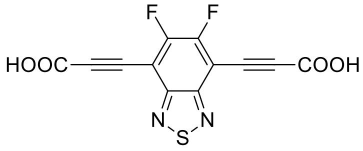

2

Under carbon dioxide atmosphere, 4,7-bis(2-trimethylsilylethinyl)-5,6-difluoro-2,1,3-benzothiadiazole (50.4 mg, 138 µmol) was dissolved in anhydrous DMSO (3 mL), caesium fluoride (51.1 mg, 336 µmol) was added and the mixture stirred vigorously at room temperature for 18 h. Subsequently, the reaction mixture was diluted with water (200 mL), extracted with dichloromethane (2 × 15 mL) and the aqueous layer acidified with 6M hydrogen chloride solution. The aqueous layer was extracted with diethyl ether (4 × 20 mL), dried over magnesium sulfate and the solvents removed in vacuo to obtain a pale orange solid (19.4 mg, 62.9 µmol, 46%).

Melting point: >200 °C (slowly decomposing)

^{13}C NMR (125 MHz, Acetone-d6, 300 K): δ = 156.9 (dd, $^{1}J_{C,F}$ = 266.1 Hz, $^{2}J_{C,F}$ = 19.5 Hz, 2C, *C*-5,6), 153.6 (2C), 150.8 (m_c, 2C), 103.8 (m_c, 2C), 93.8 (m_c, 2C), 73.2 (m_c, 2C) ppm;

IR (ATR): ṽ = 3518 (m_{br}), 2895 (m_{br}), 2230 (s), 1671 (vs), 1499 (m), 1451 (vs), 1400 (vs), 1347 (s), 1314 (vs), 1242 (vs), 1165 (s), 1017 (vs), 890 (s), 849 (s), 828 (s), 742 (s) cm^{-1};

MS (EI, 70 eV): m/z (%) = 220 (100) $[M-2CO_2]^+$, 193 (11) $[M-2CO_2-2C]$.

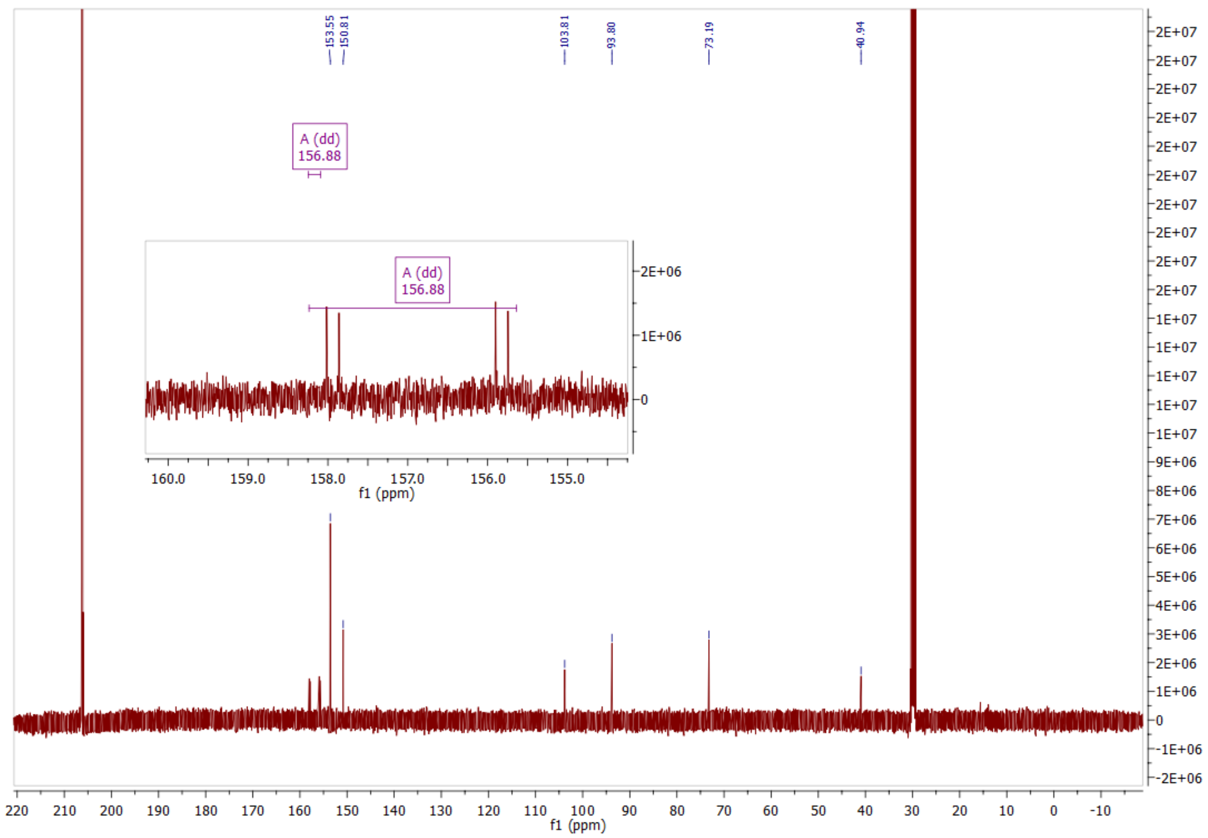

Figure S7: ^{13}C NMR spectrum of compound **2** measured in deuterated acetone.

Synthesis of 3,3'-(5,6-Dicyano-2,1,3-benzothiadiazole-4,7-diyl)dipropiolic acid (3)

4,7-Bis-(2-trimethylsilylethinyl)-5,6-dicyano-2,1,3-benzothiadiazole (12a)

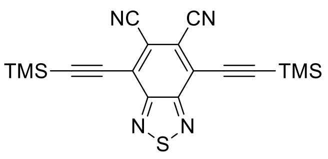

12a

a) Via Sonogashira reaction: Under nitrogen atmosphere, 5,6-dicyano-4,7-dibromo-2,1,3-benzothiadiazole (**11a**, 158 mg, 459 µmol), bis(triphenylphosphino)dichloropalladium(II) (32.9 mg, 46.8 µmol) and copper(I) iodide (13.0 mg, 68.3 µmol) and triphenylphosphine (16.3 mg, 62.1 µmol) were dissolved in abs. THF (10 mL) and diisopropylamine (10 mL) was added. Subsequently, trimethylsilylacetylene (160 µL, 1.14 mmol) was added and the solution was

stirred for 18 h at room temperature. The reaction mixture was filtered through a bed of celite, washed with dichloromethane and the solvents evaporated in vacuo. The obtained crude product was purified by column chromatography using silica and dichloromethane/*n*-pentane (1:2, R_f = 0.21) as eluent to obtain a yellow solid (17.6 mg, 46.5 µmol, 10 %).

b) Via Stille reaction: Under nitrogen atmosphere, 5,6-dicyano-4,7-diiodo-2,1,3-benzothiadiazole (**11b**, 359 mg, 820 µmol) and bis(tri-*tert*-butylphosphino)palladium(0) (43.6 mg, 85.3 µmol) and 1-trimethylsilyl-2-tributylstannylacetylen (0.66 mL, 1.80 mmol)) were dissolved in anhydrous toluene (20 mL) and stirred at 110 °C for 18 h. Subsequently, the mixture was filtered over a bed of celite, the solvent removed in vacuo and the crude product purified by column chromatography using silica and dichloromethane/*n*-pentane (3:2, R_f = 0.50) as eluent to give yellow needles (278 mg, 734 µmol, 90 %).

Melting point: 260 °C;

^{1}H NMR (500MHz, $CDCl_3$, 300 K): δ = 0.38 (s, 18H) ppm;

^{13}C NMR (125 MHz, $CDCl_3$, 300K): δ = 153.9 (2C), 124.0 (2C), 117.3 (2C), 116.6 (2C), 113.9 (2C), 96.3 (2C), -0.4 (6C) ppm;

IR (ATR): $\tilde{\upsilon}$ = 2966 (w), 2905 (w), 2231 (w), 2166 (w), 1543 (w), 1458 (m), 1361 (m), 1243 (m), 1138 (w), 957 (s), 841 (vs), 761 (vs), 710 (m), 666 (s) cm^{-1};

MS (EI, 70 eV): m/z (%) = 378 (27) $[M]^+$, 363 (100) $[M-Me]^+$;

HR-MS (EI, 70 eV): m/z = calcd. for $C_{18}H_{18}N_4SSi_2$ $[M]^+$ 378.0791, found 378.0789.

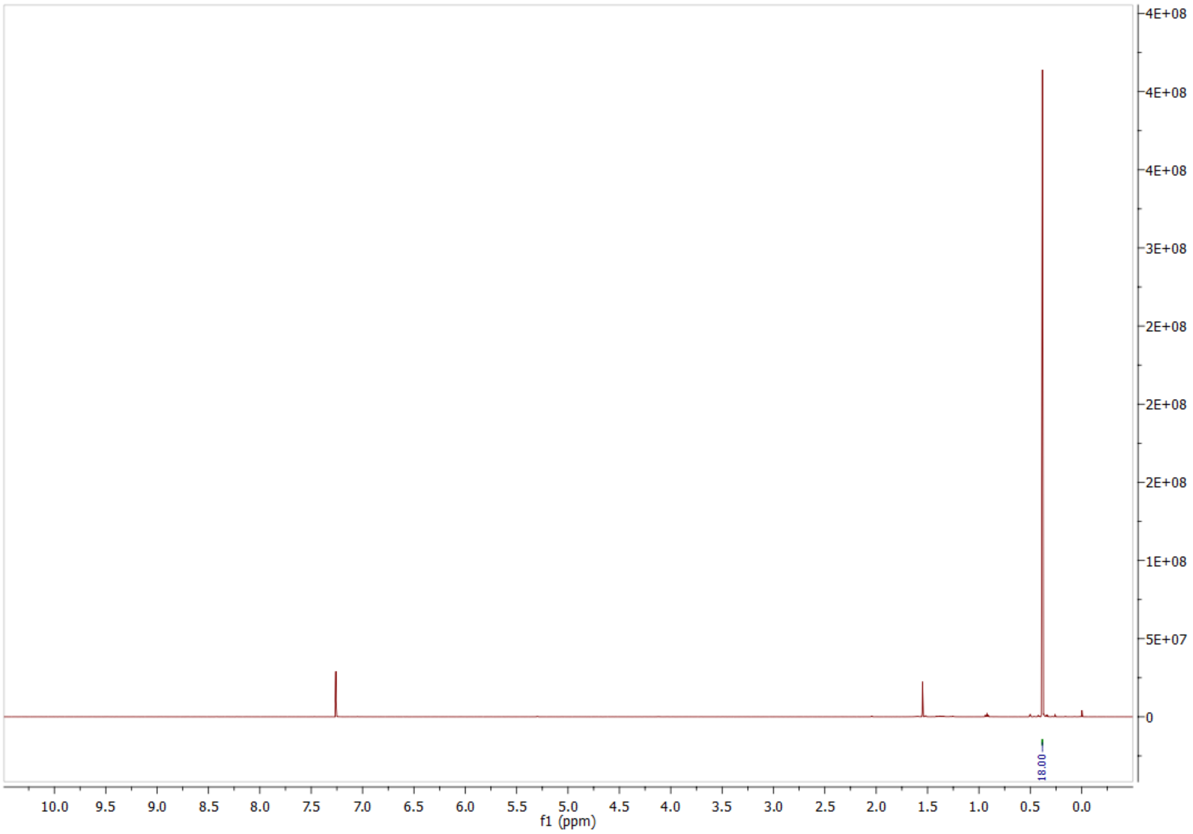

Figure S8: ^{1}H NMR spectrum of compound **12a** measured in deuterated chloroform.

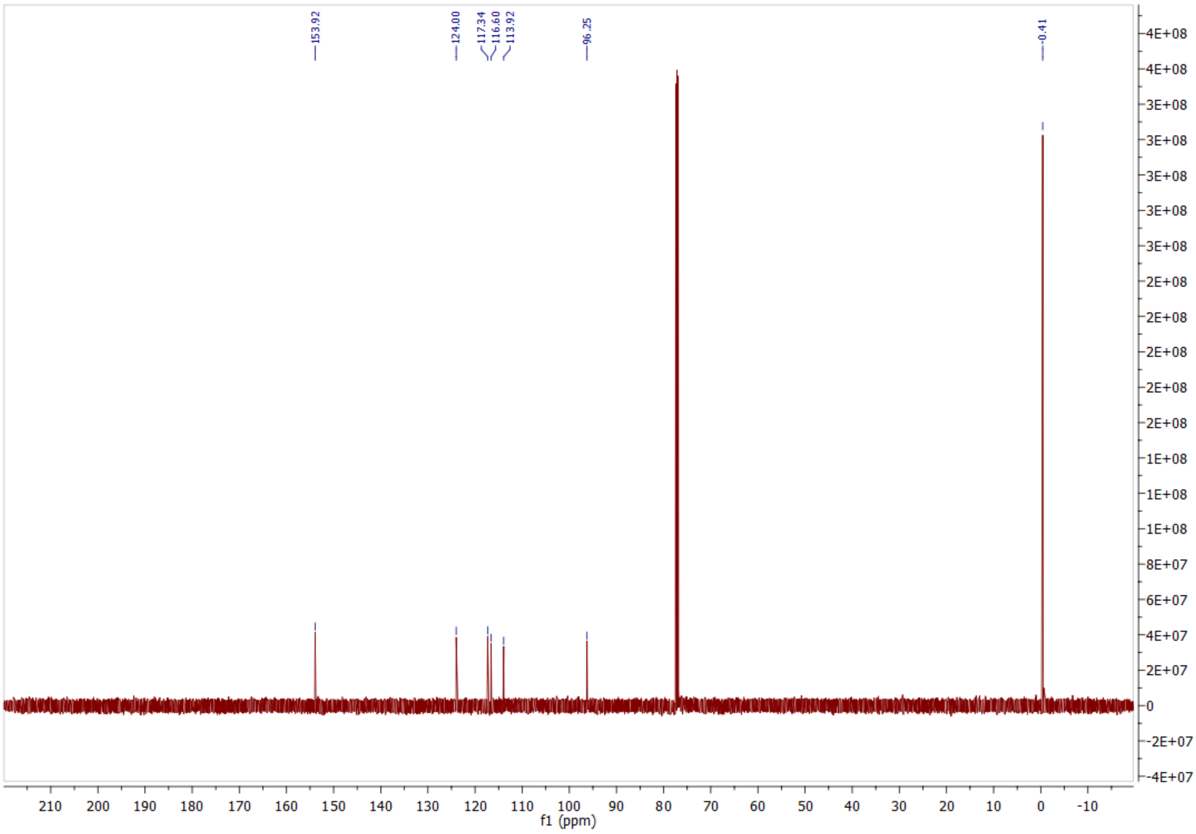

Figure S9: ^{13}C NMR spectrum of compound **12a** measured in deuterated chloroform.

4,7-Bis(2-triisopropylsilylethinyl)-5,6-dicyano-2,1,3-benzothiadiazole (12b)

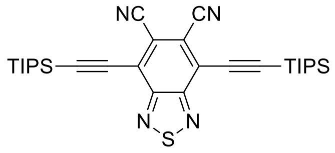

12b

Under nitrogen atmosphere 5,6-dicyano-4,7-diiodo-2,1,3-benzothiadiazole (**11b**, 148 mg, 338 µmol), bis-(triphenylphosphino)dichloropalladium(II) (24.9 mg, 35.5 µmol) and copper(I) iodide (9.80 mg, 51.5 µmol) and triphenylphosphine (10.0 mg, 38.1 µmol) were dissolved in abs. THF (7 mL) and diisopropylamine (7 mL) was added. Subsequently, triisopropylsilylacetylene (230 µL, 1.03 mmol) was added and the solution was stirred for 18 h at room temperature. The reaction mixture was filtered through a bed of celite, washed with dichloromethane and the solvents evaporated in vacuo. The obtained crude product was purified by column chromatography using silica and dichloromethane/*n*-pentane (1:3, R_f = 0.23) as eluent to obtain a yellow solid (38.4 mg, 70.2 µmol, 21%).

^{1}H NMR (500MHz, $CDCl_3$, 300 K): δ = 1.31-1.08 (m, 42H) ppm;

^{13}C NMR (125 MHz, $CDCl_3$, 300 K): δ = 154.4 (2C), 124.2 (2C), 116.9 (2C), 114.1 (2C), 114.0 (2C), 98.2 (2C), 18.7 (12C), 11.3 (6C) ppm;

MS (EI, 70 eV): m/z (%) = 546 (8) $[M]^+$, 503 (100) $[M-^{i}Pr]^+$;

HR-MS (EI, 70 eV): m/z = calcd. for $C_{30}H_{42}N_4SSi_2$ $[M]^+$ 546.2669, found 546.2668.

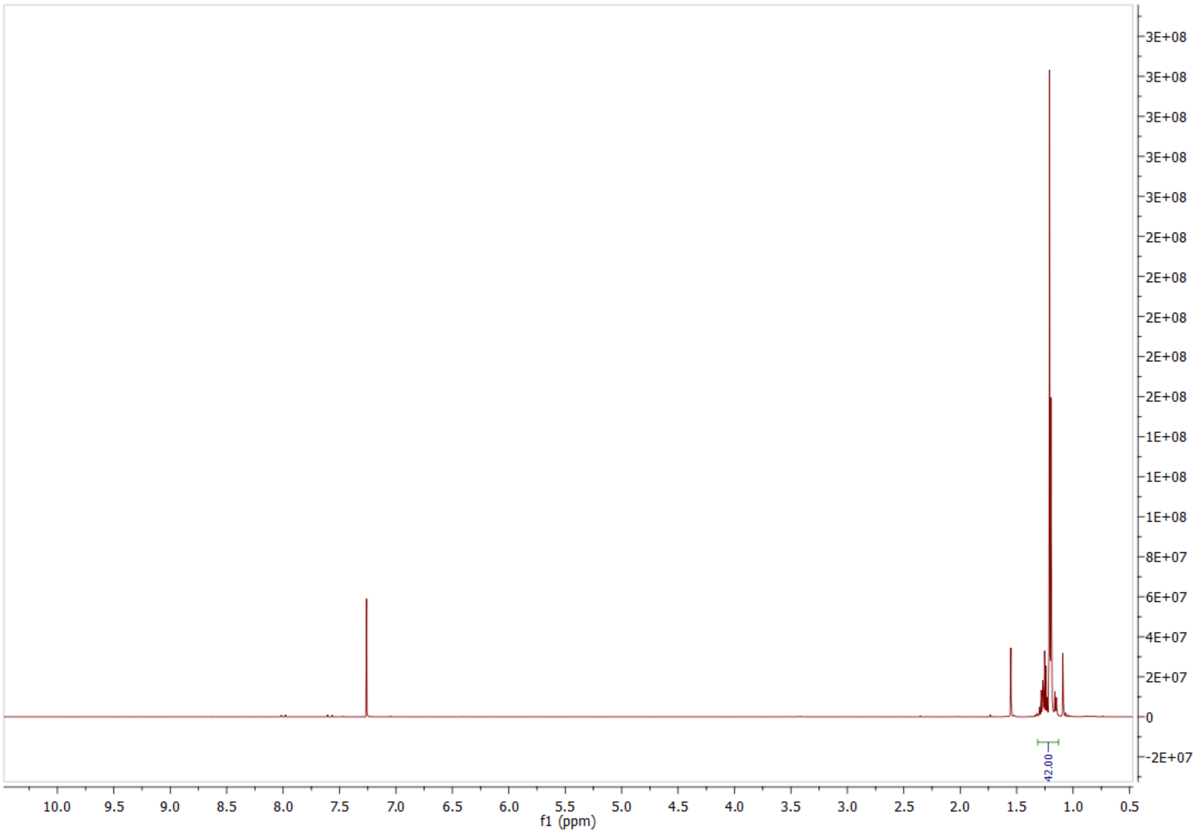

Figure S10: ^{1}H NMR spectrum of compound **12b** measured in deuterated chloroform.

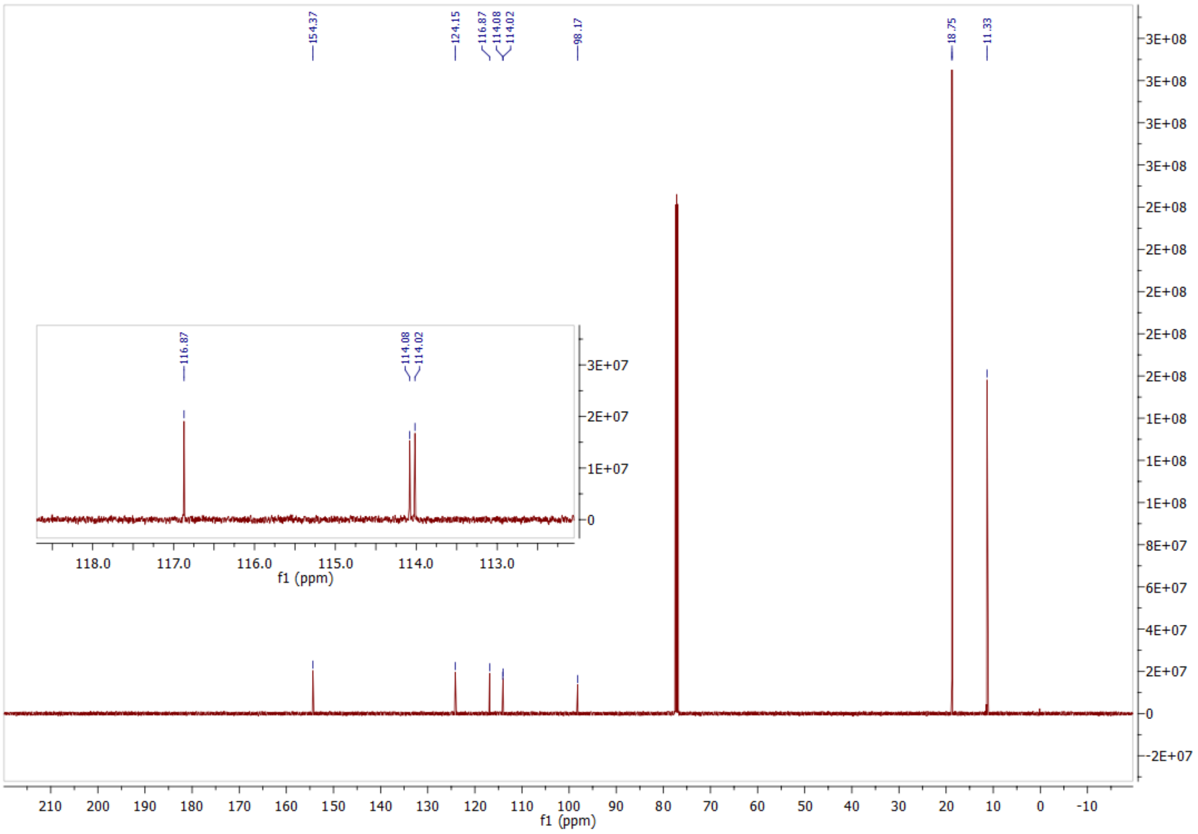

Figure S11: ^{13}C NMR spectrum of compound **12b** measured in deuterated chloroform.

3,3'-(5,6-Dicyano-2,1,3-benzothiadiazole-4,7-diyl)dipropiolic acid (3)

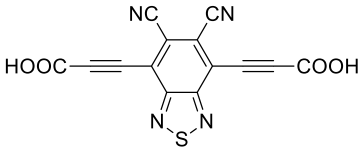

3

Under carbon dioxide atmosphere, 4,7-bis(2-trimethylsilylethinyl)-5,6-dicyano-2,1,3-benzothiadiazole (**12a**, 31.2 mg, 82.4 µmol) was dissolved in anhydrous MeCN (8 mL) and cooled to 0 °C. Caesium fluoride (48.0 mg, 316 µmol) was added and the mixture stirred vigorously at 0 °C for 3 h while bubbling carbon dioxide through the reaction mixture. Subsequently, the reaction mixture was diluted with water (50 mL), extracted with dichloromethane (2 × 10 mL) and the aqueous layer acidified with 6M hydrogen chloride solution while cooled to 0 °C. The aqueous layer was extracted with diethylether (4 × 15 mL), dried over magnesium sulfate and the solvents removed in vacuo to obtain brown solid (24.0 mg, 74.5 µmol, 90%).

Melting point: >200 °C (slowly decomposing);

^{13}C NMR (125 MHz, Acetone-d6, 300 K): δ = 154.9 (2C), 153.1 (2C), 123.1 (2C), 119.2 (2C), 114.4 (2C), 95.0 (2C), 76.2 (2C) ppm;

IR (ATR): ῦ = 2913 (m_{br}), 2229 (s), 1703 (vs), 1476 (s), 1464 (s), 1377 (s), 1342 (s), 1221 (vs), 1144 (s), 997 (s), 886 (s), 856 (s), 813 (s), 754 (s), 741 (s), 629 (vs), 602 (s), 546 (vs), 498 (s), 417 (s), 408 s) cm^{-1};

MS (EI, 70 eV): m/z (%) = 220 (100) [M-2CO_2]$^+$, 207 (13) [M-CO_2-CCO_2]$^+$.

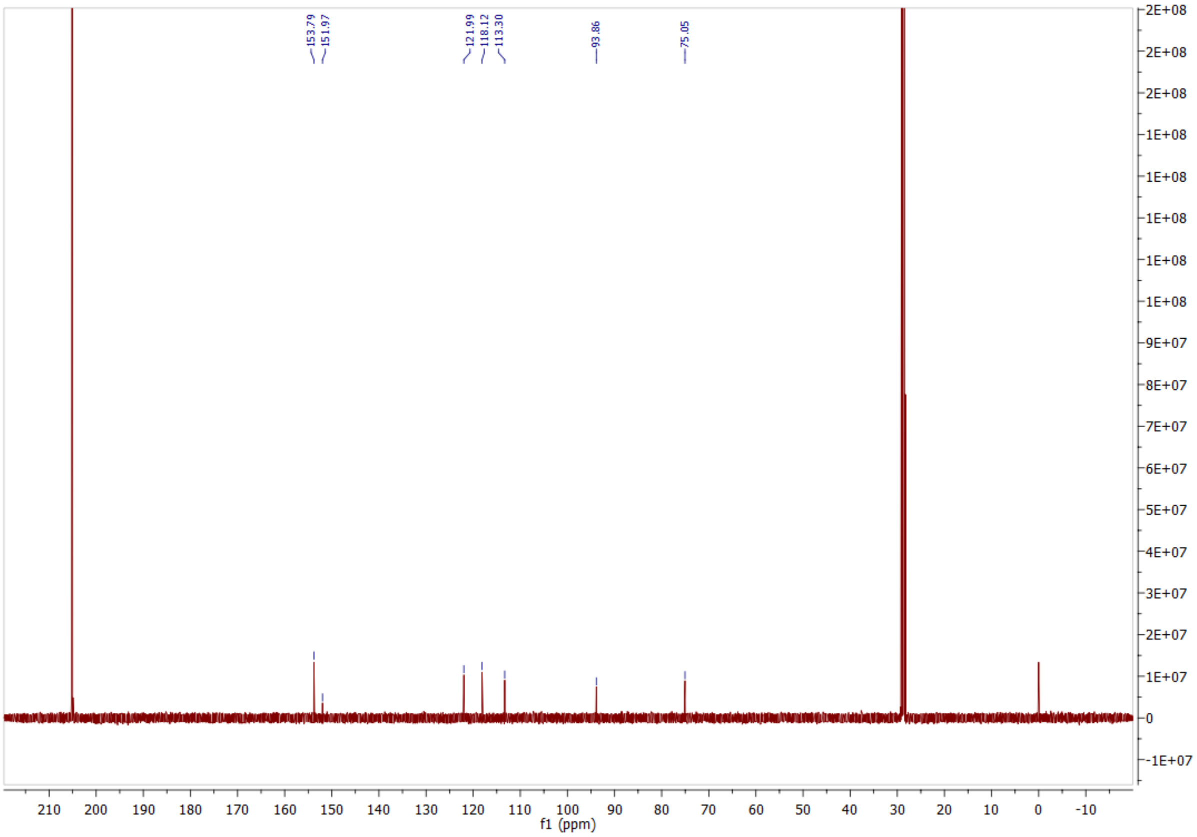

Figure S12: ^{13}C NMR spectrum of compound **3** measured in deuterated acetone.

Synthesis of 3,3'-(5,6-dicyano-1,3-benzodioxole-4,7-diyl)dipropiolic acid (4)

5,6-Dicyano-1,3-benzodioxole (14a)

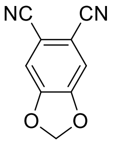

14a

Under nitrogen atmosphere, 5,6-Dibromo-1,3-benzodioxole (**13a**, 1.58 g, 5.64 mmol), zinc(II) cyanide (670 mg, 5.71 mmol) and [1,1'-bis(diphenylphosphino)ferrocene]dichloro-palladium(II) (208 mg, 284 µmol) were dissolved in anhydrous dimethylacetamide (12 mL) and stirred for 3 h at 150 °C. Subsequently, the mixture was diluted with ethyl acetate (50 mL), washed with water (2 × 50 mL), the organic layer dried over magnesium sulfate and the solvent removed in vacuo. The crude product was purified by column chromatography using silica and dichloromethane as eluent (R_f = 0.50) to obtain a colourless solid (829 mg, 4.82 mmol, 85%).

^{1}H NMR (500MHz, $CDCl_3$, 300 K): δ = 7.14 (s, 2H), 6.21 (s, 2H) ppm;

^{13}C NMR (125 MHz, $CDCl_3$, 300 K): δ = 151.7 (2C), 115.5 (2C), 112.8 (2C), 111.0 (2C), 104.0 ppm;

MS (EI, 70 eV): m/z (%) = 172 (100) $[M]^+$;

HR-MS (EI, 70 eV): m/z = calcd. for $C_9H_4N_2O_2$ $[M]^+$ 172.0267, found 172.0273.

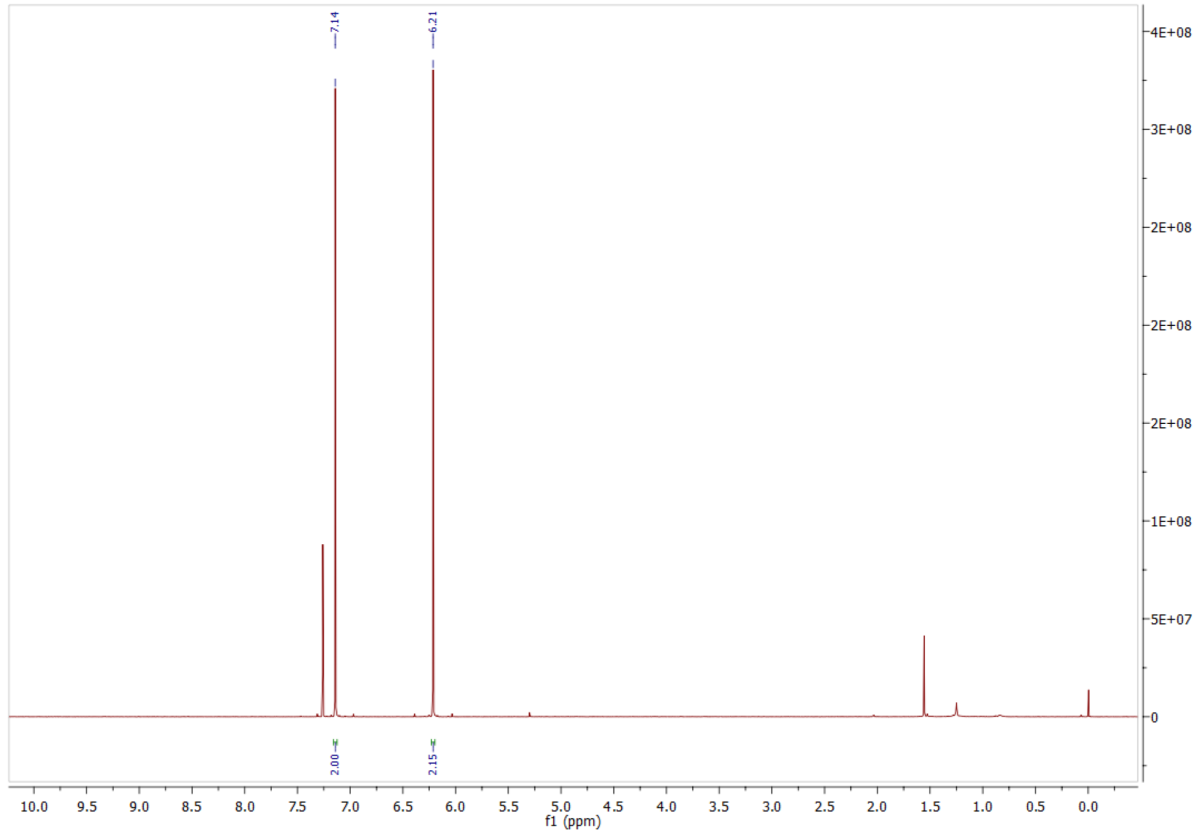

Figure S13: ^{1}H NMR spectrum of compound **14a** measured in deuterated chloroform.

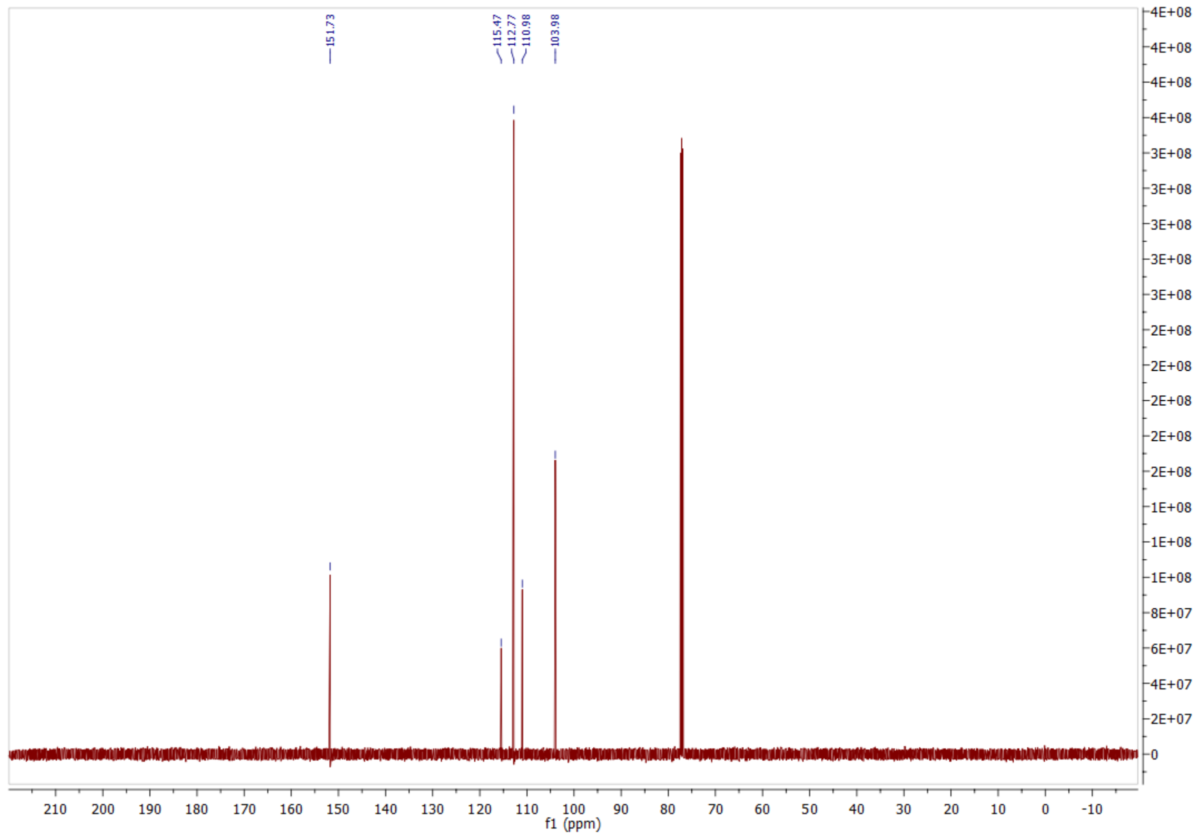

Figure S14: ^{13}C NMR spectrum of compound **14a** measured in deuterated chloroform.

4,7-Dibromo-5,6-dicyano-1,3-benzodioxole (15a)

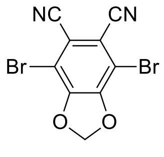

15a

Dibromoisocyanuric acid (1.63 g, 5.69 mmol) was dissolved in 5% fuming sulfuric acid and stirred for 30 min under ice cooling. 5,6-Dicyano-1,3-benzodioxole (**14a**, 490 mg, 2.85 mmol) was added and the mixture stirred for 10 min while maintaining the cooling. Subsequently, the mixture was poured into ice water (600 mL), extracted with diethyl ether (3 × 150 mL), washed with sat. sodium thiosulfate and sat. sodium bicarbonate solutions (150 mL each), dried over magnesium sulfate and the solvent removed in vacuo. The crude product was purified by column chromatography using silica and dichloromethane/*n*-pentane (2:1, R_f = 0.48) to obtain a colourless solid (823 mg, 2.49 mmol, 88%).

Melting point: 240 °C

^{1}H NMR (500MHz, $CDCl_3$, 300 K): δ = 6.35 (s, 2H) ppm;

^{13}C NMR (125 MHz, $CDCl_3$, 300 K): δ = 150.2 (2C), 115.5 (2C), 113.6 (2C), 104.4 (2C), 104.1 ppm;

IR (ATR): ῦ = 3003 (w), 2949 (w), 2925 (w), 2649 (w), 2232 (s), 1586 (s), 1533 (w), 1494 (s), 1446 (vs), 1392 (s), 1346 (m), 1286 (vs), 1201 (s), 1117 (m), 1062 (s), 1046 (vs), 926 (vs), 845 (m), 804 (s), 728 (s) cm^{-1};

MS (EI, 70 eV): m/z (%) = 329 (100) $[M]^+$;

HR-MS (EI, 70 eV): m/z = calcd. for $C_9H_2Br_2N_2O_2$ $[M]^+$ 329.8456, found 329.8463.

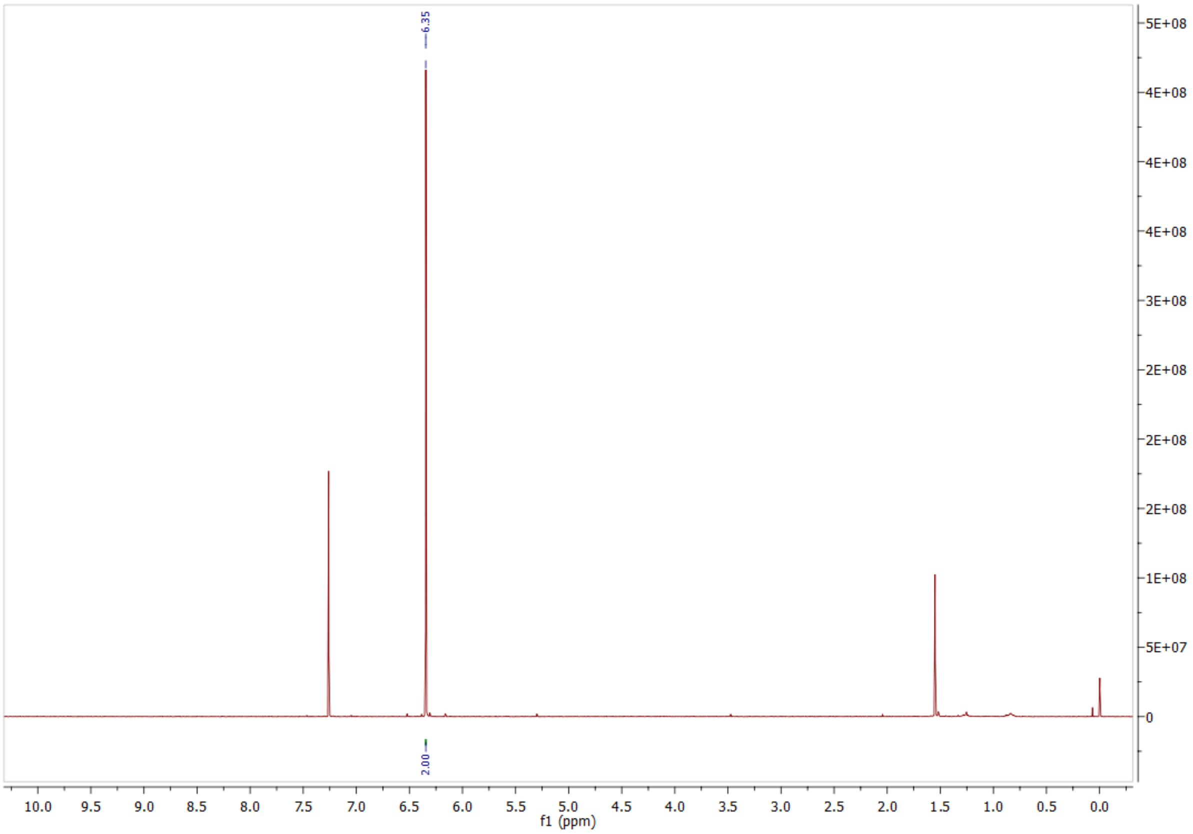

Figure S15: ^{1}H NMR spectrum of compound **15a** measured in deuterated chloroform.

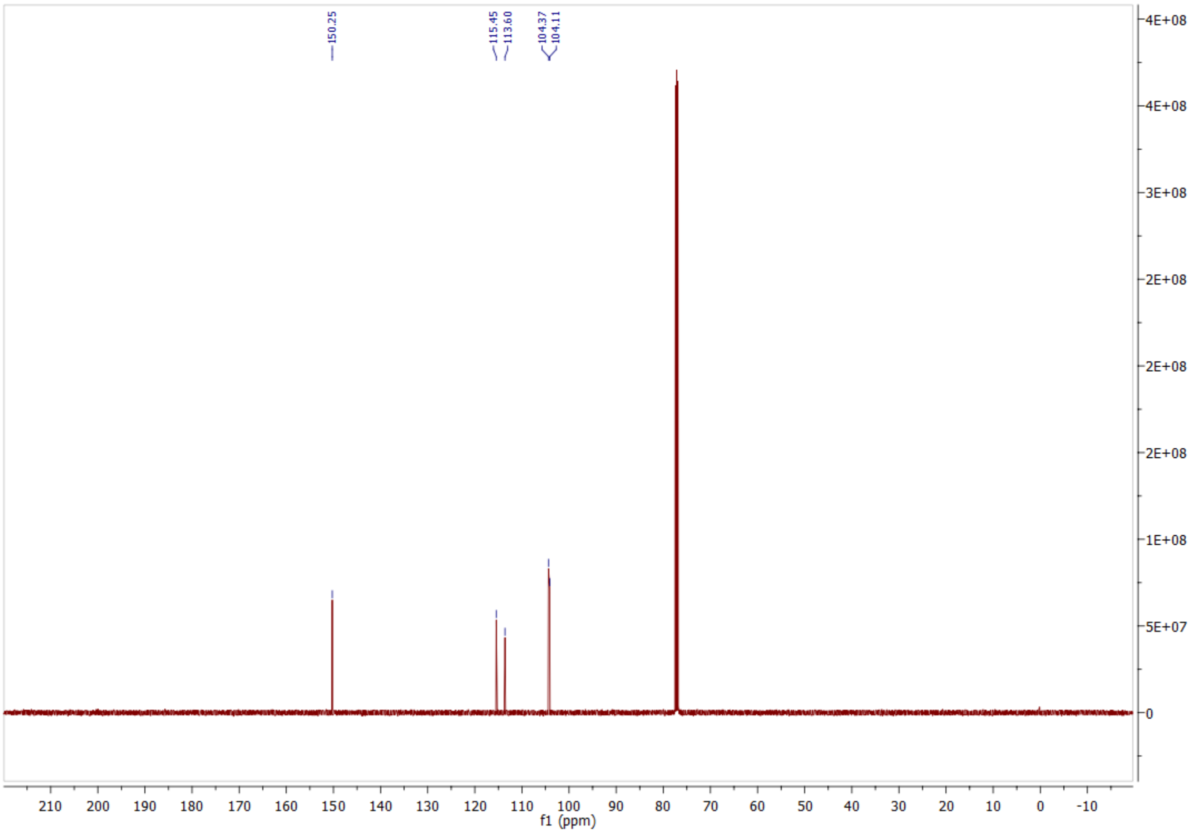

Figure S16: ^{13}C NMR spectrum of compound **15a** measured in deuterated chloroform.

4,7-Bis-(2-trimethylsilylethinyl)-5,6-dicyano-1,3-benzodioxole (16a)

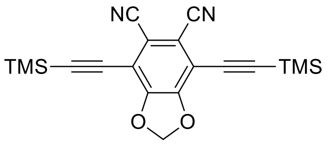

16a

Under nitrogen atmosphere, 4,7-dibromo-5,6-dicyano-1,3-benzodioxole (**15a**, 378 mg, 1.15 mmol) and bis(tri-*tert*-butylphosphino)palladium(0) (58.0 mg, 113 µmol) and 1-trimethylsilyl-2-tributylstannylacetylene (0.91 mL, 2.53 mmol)) were dissolved in anhydrous toluene (20 mL) and stirred at 110 °C for 18 h. Subsequently, the mixture was filtered over a bed of celite, the solvent removed in vacuo and the crude product purified by column chromatography using silica and dichloromethane/*n*-pentane (3:2, R_f = 0.56) as eluent. The obtained solid was recrystallized from ethanol to give colorless needles (336 mg, 921 µmol, 80%).

Melting point: 225 °C

^{1}H NMR (500MHz, $CDCl_3$, 300 K): δ = 6.29 (s, 2H), 0.29 (s, 18H) ppm;

^{13}C NMR (125 MHz, $CDCl_3$, 300 K): δ = 152.0 (2C), 114.5 (2C), 113.9 (2C), 110.3 (2C), 108.8 (2C), 104.6 (2C), 92.7, 0.3 (6C) ppm;

IR (ATR): ῦ = 3009 (w), 2967 (m), 2228 (m), 1575 (m), 1498 (m), 1438 (vs), 1422 (s), 1275 (s), 1256 (s), 1245 (s), 1144 (s), 1110 (m), 1065 (m), 1042 (s), 971 (s), 913 (m), 840 (vs), 761 (vs), 709 (s), 667 (s) cm^{-1};

MS (EI, 70 eV): m/z (%) = 364 (15) $[M]^+$, 349 (100) $[M-Me]^+$;

HR-MS (EI, 70 eV): m/z = calcd. for $C_{19}H_{20}N_2O_2Si_2$ $[M]^+$ 364.1063, found 364.1063.

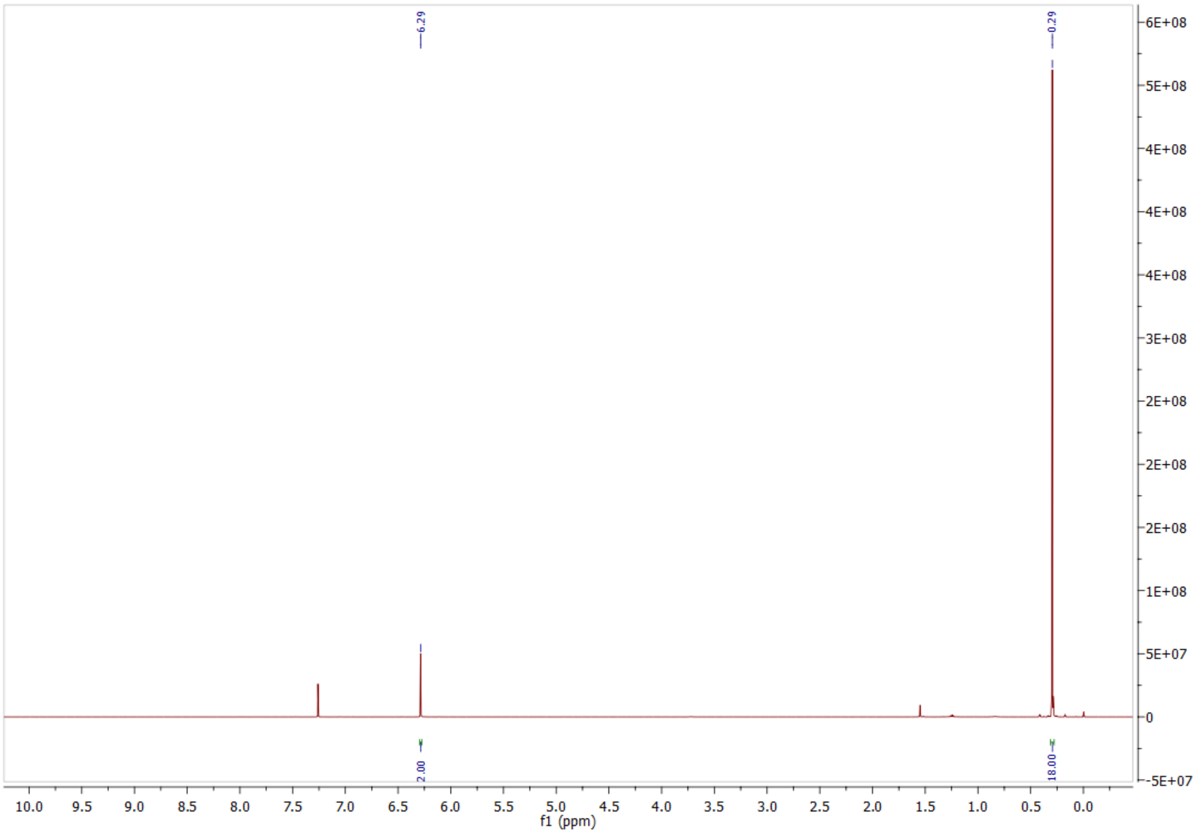

Figure S17: ^{1}H NMR spectrum of compound **16a** measured in deuterated chloroform.

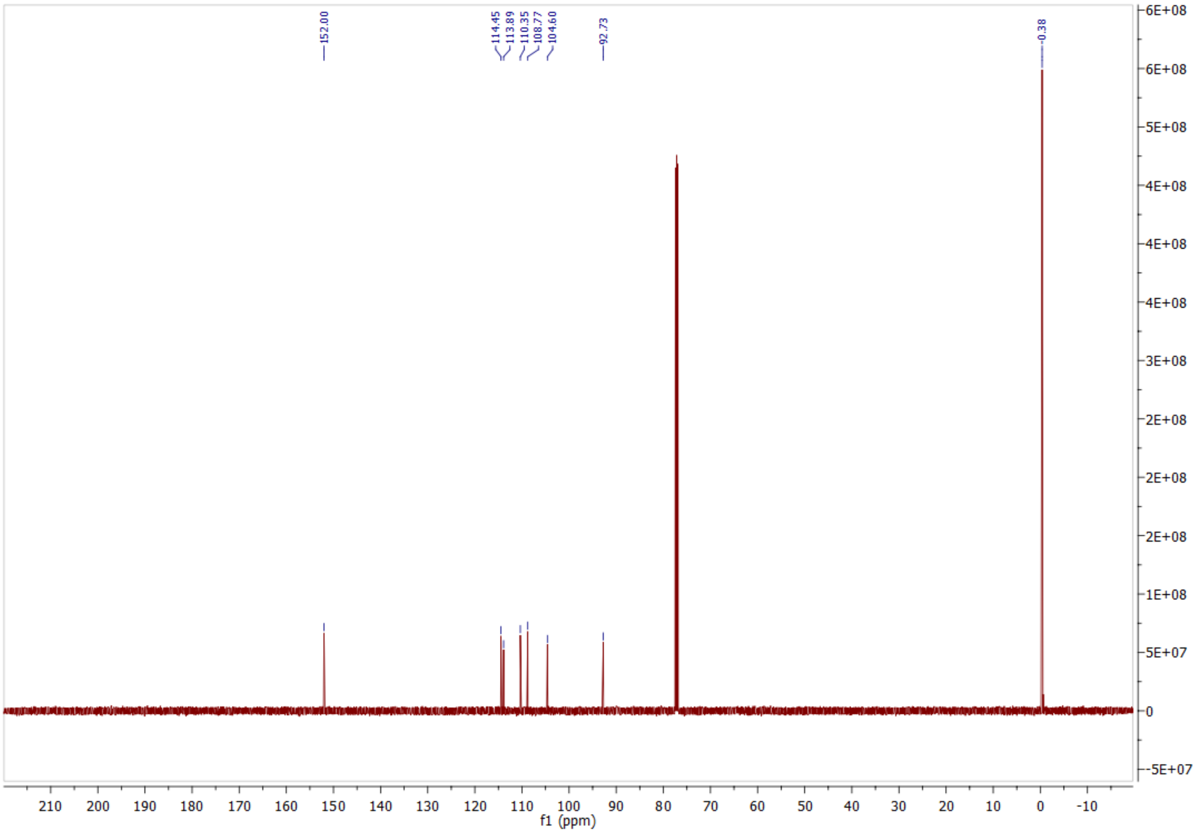

Figure S18: ^{13}C NMR spectrum of compound **16a** measured in deuterated chloroform.

3,3'-(5,6-Dicyano-1,3-benzodioxole-4,7-diyl)dipropiolic acid (4)

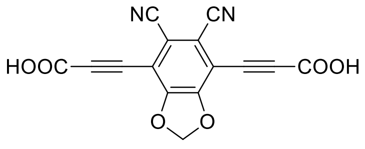

4

Under carbon dioxide atmosphere, 4,7-bis(2-trimethylsilylethinyl)-5,6-dicyano-1,3-benzodioxole (**16a**, 616 mg, 1.69 mmol) was dissolved in anhydrous DMSO (8 mL), caesium fluoride (646 mg, 4.25 mmol) was added and the mixture stirred vigorously at room temperature for 18 h. Subsequently, the reaction mixture was diluted with water (400 mL), extracted with dichloromethane (3 × 150 mL) and the aqueous phase acidified with 6 M hydrogen chloride solution. The aqueous layer was extracted with diethyl ether (5 × 50 mL), the organic layer washed with 2 M hydrogen chloride solution (200 mL), dried over magnesium sulfate and the solvents removed in vacuo to obtain a pale brownish solid (450 mg, 1.46 mmol, 86%).

Melting point: >200 °C (slowly decomposing)

^{1}H NMR (500MHz, Acetone-d6, 300 K): δ = 6.67 (s, 2H), ppm;

^{13}C NMR (125 MHz, Acetone-d6, 300 K): δ = 156.2 (2C), 153.2 (2C), 114.8 (2C), 114.2 (2C), 108.2 (2C), 105.8 (2C), 91.5 (2C), 73.7 ppm;

IR (ATR): ῦ = 3561 (m), 2895 (m_{br}), 2229 (s), 1709 (s), 1671 (s), 1495 (s), 1449 (vs), 1389 (s), 1332 (s), 1267 (s), 1221 (vs), 1167 (s), 1042 (vs), 917 (s), 813 (s), 745 (s) cm^{-1};

MS (EI, 70 eV): m/z (%) = 220 (100) $[M-2CO_2]^+$.

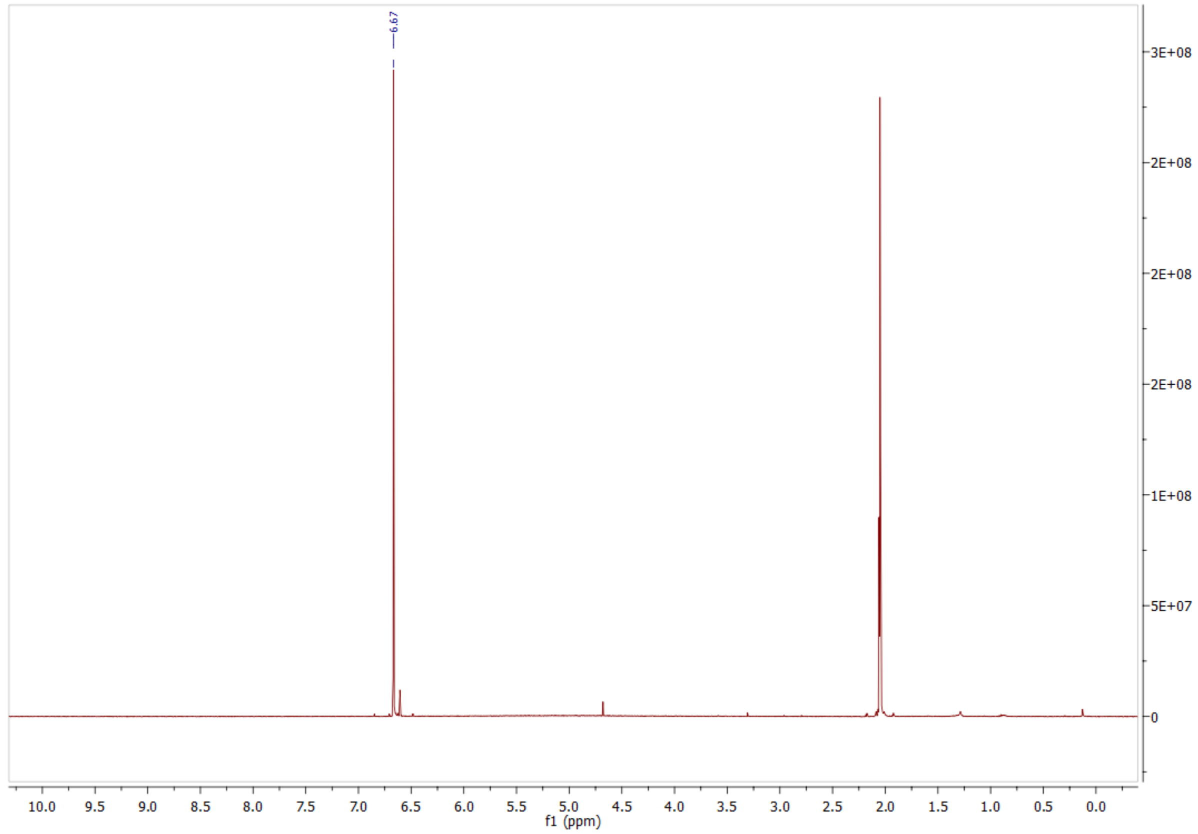

Figure S19: ^{1}H NMR spectrum of compound **4** measured in deuterated acetone.

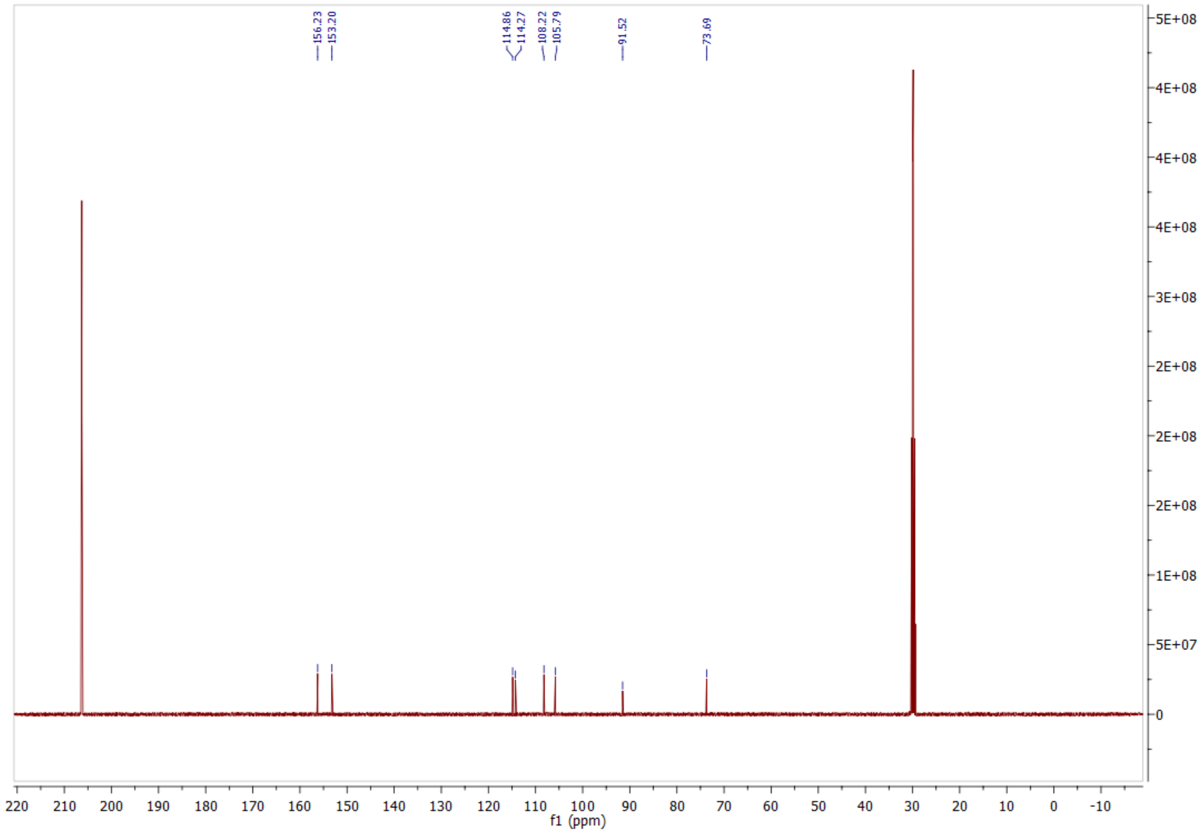

Figure S20: ^{13}C NMR spectrum of compound **4** measured in deuterated acetone.

Synthesis of 3,3'-(6,7-dicyano-1,4-benzodioxole-5,8-diyl)dipropiolic acid (5)

6,7-Dicyano-1,4-benzodioxane (14b)

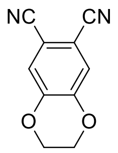

14b

Under nitrogen atmosphere, 6,7-dibromo-1,4-benzodioxane (**13b**, 1.77 g, 6.02 mmol), zinc(II) cyanide (1.48 g, 12.3 mmol) and [1,1′-bis(diphenylphosphino)ferrocene]dichloro-palladium(II) (225 mg, 308 µmol) were dissolved in anhydrous dimethylacetamide (12 mL) and stirred for 4 h at 150 °C. Subsequently, the mixture was diluted with ethyl acetate (50 mL), washed with water (2 × 50 mL), the organic layer dried over magnesium sulfate and the solvent removed in vacuo. The crude product was purified by column chromatography using silica and dichloromethane as eluent (R_f = 0.63) to obtain a colorless solid (714 mg, 3.84 mmol, 64%).

Melting point: 234 °C

^{1}H NMR (500MHz, DMSO-d6, 300 K): δ = 7.73 (s, 2H), 4.40 (s, 4H) ppm;

^{13}C NMR (125 MHz, DMSO-d6, 300 K): δ = 147.7 (2C), 123.0 (2C), 115.8 (2C), 107.2 (2C), 64.6 (2C) ppm;

IR (ATR): ῦ = 3118 (w), 3049 (m), 3002 (w), 2948 (w) 2888 (w), 2230 (w), 2659 (w), 2230 (s), 1792 (w), 1602 (m), 1562 (s), 1504 (s), 1454 (s), 1316 (s), 1238 (s), 1204 (s), 1185 (m), 1100 (m), 1059 (s), 1008 (m), 919 (s), 900 (vs), 750 (m), 718 (vs), 660 (m) cm^{-1};

MS (EI, 70 eV): m/z (%) = 186 (100) [M]$^+$;

HR-MS (EI, 70 eV): m/z = calcd. for $C_{10}H_6N_2O_2$ [M]$^+$ 186.0429, found 186.0427.

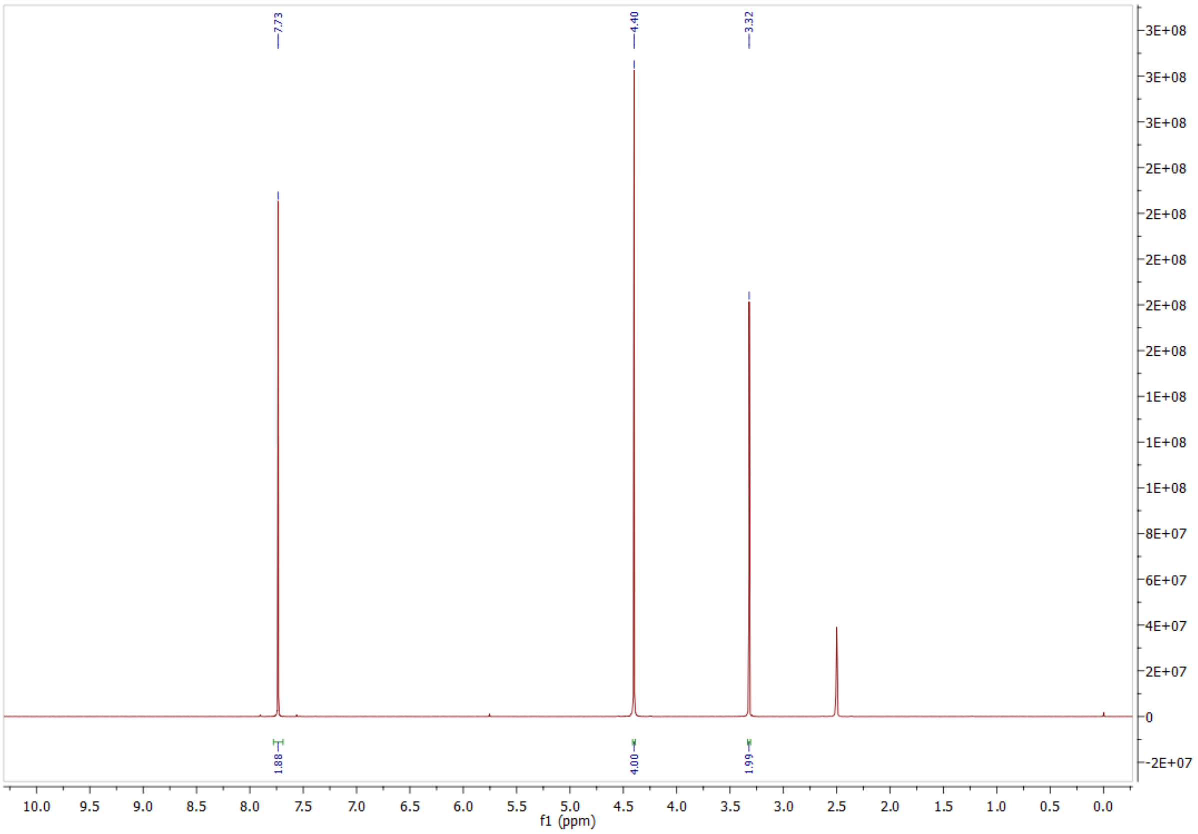

Figure S21: ^{1}H NMR spectrum of compound **14b** measured in deuterated DMSO.

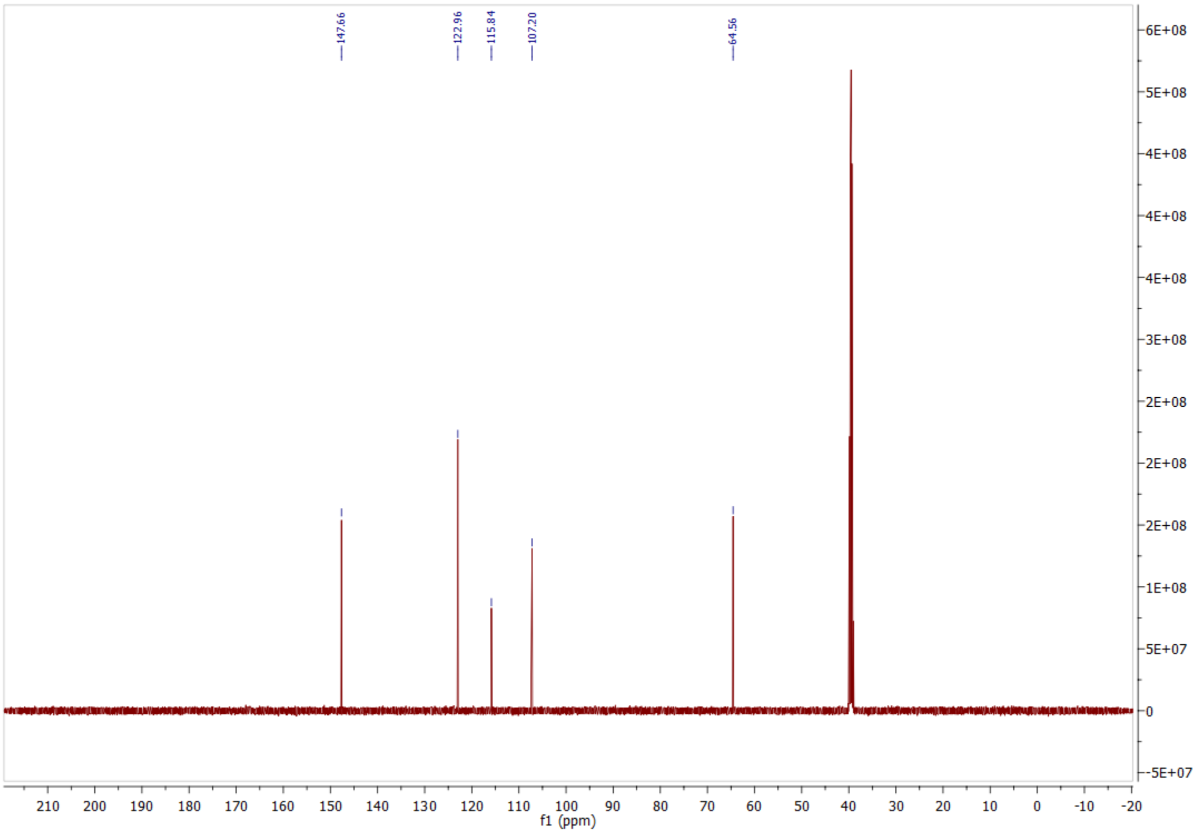

Figure S22: ^{13}C NMR spectrum of compound **14b** measured in deuterated DMSO.

5,8-Dibromo-6,7-dicyano-1,4-benzodioxane (15b)

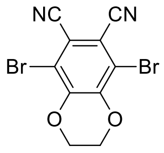

15b

Dibromoisocyanuric acid (1.90 g, 6.64 mmol) was dissolved in 5% fuming sulfuric acid and stirred for 30 min under ice cooling. 6,7-Dicyano-1,4-benzodioxane (**14b**, 618 mg, 3.32 mmol) was added and the mixture stirred for 10 min while maintaining the cooling. Subsequently, the mixture was poured into ice water (900 mL), extracted with diethyl ether (4 × 200 mL), washed with sat. sodium thiosulfate and sat. sodium bicarbonate solutions (200 mL each), dried over magnesium sulfate and the solvent removed in vacuo. The crude product was purified by column chromatography using silica and dichloromethane/*n*-pentane (2:1, R_f = 0.51) to obtain a colorless solid (534 mg, 1.55 mmol, 47%).

Melting point: 311 °C

^{1}H NMR (500MHz, $CDCl_3$, 300 K): δ = 4.51 (s, 4H) ppm;

^{13}C NMR (125 MHz, $CDCl_3$, 300 K): δ = 145.6 (2C), 115.4 (2C), 114.0 (2C), 113.3 (2C), 65.4 (2C) ppm;

IR (ATR): ṽ = 2947 (w), 2235 (s), 1771 (m), 1566 (m), 1535 (s), 1463 (s), 1436 (s), 1424 (s), 1324 (vs), 1243 (s), 1209 (s), 1108 (s), 1070 (vs), 1008 (s), 931 (vs), 879 (m), 845 (m), 817 (m), 768 (s), 730 (s), 665 (m) cm^{-1};

MS (EI, 70 eV): m/z (%) = 343 (100) $[M]^+$, 234 (34) $[M-BrC_2H_4]^+$;

HR-MS (EI, 70 eV): m/z = calcd. for $C_{10}H_4Br_2N_2O_2$ $[M]^+$ 343.8619, found 343.8614.

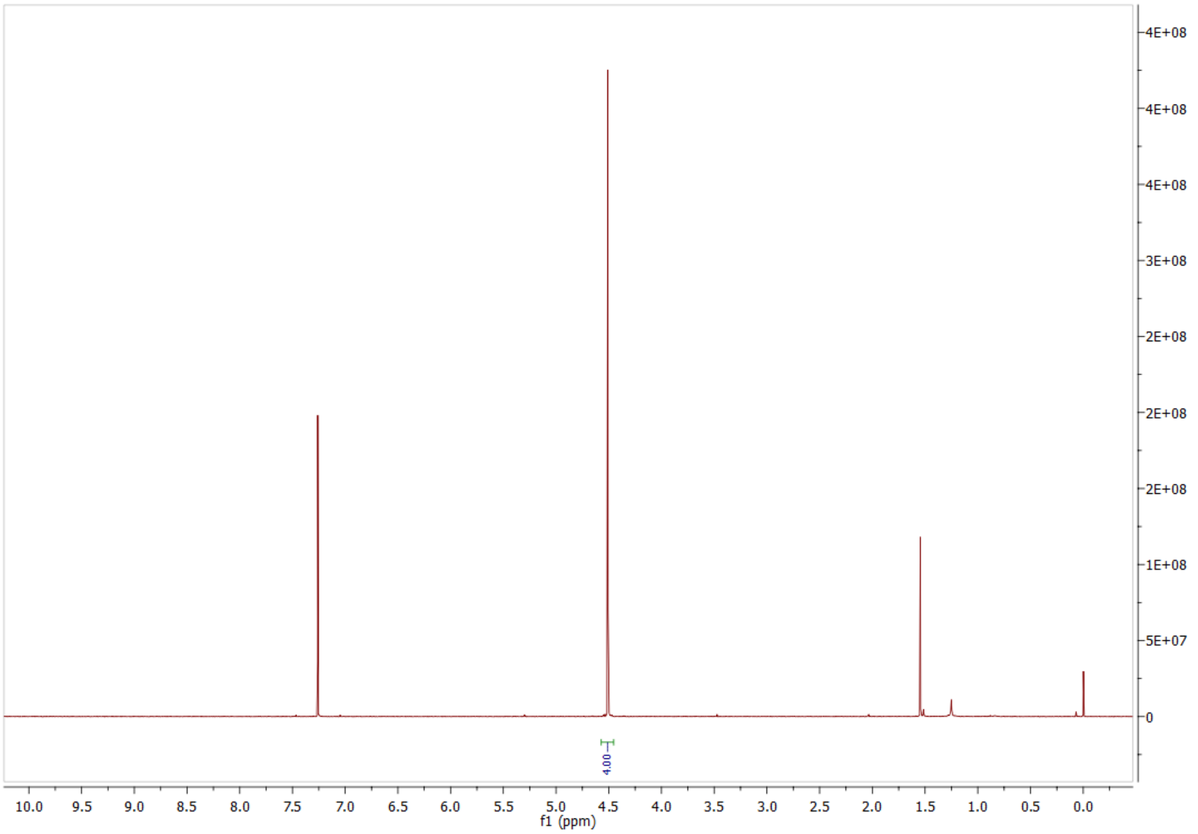

Figure S23: ^{1}H NMR spectrum of compound **15b** measured in deuterated chloroform.

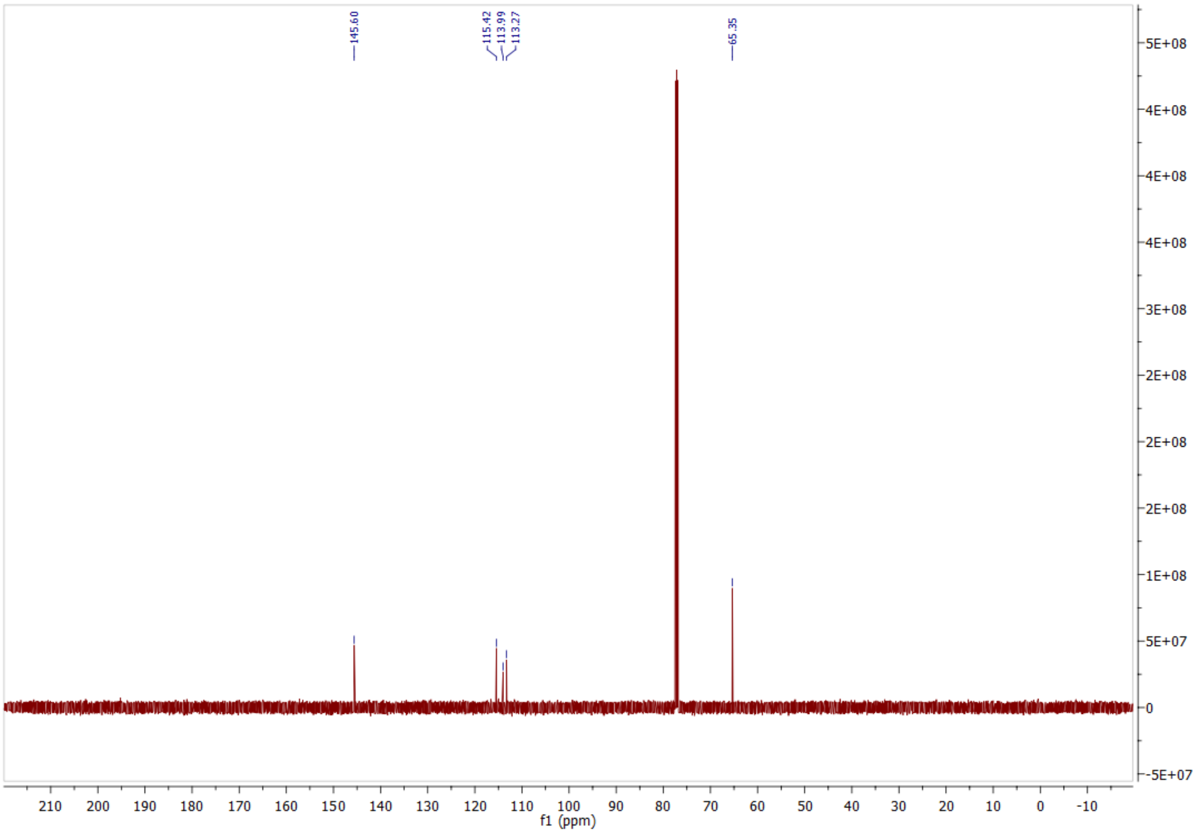

Figure S24: ^{1}H NMR spectrum of compound **15b** measured in deuterated chloroform.

5,8-Bis-(2-trimethylsilylethinyl)-6,7-dicyano-1,4-benzodioxane (16b)

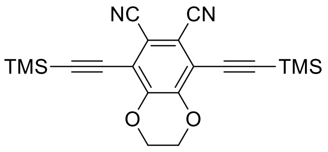

16b

Under nitrogen atmosphere, 5,8-dibromo-6,7-dicyano-1,4-benzodioxane (**15b**, 463 mg, 1.35 mmol) and bis(tri-*tert*-butylphosphino)palladium(0) (71.0 mg, 139 µmol) and 1-trimethylsilyl-2-tributylstannylacetylene (1.01 mL, 2.83 mmol)) were dissolved in anhydrous toluene (20 mL) and stirred at 110 °C for 18 h. Subsequently, the mixture was filtered over a bed of celite, the solvent removed in vacuo and the crude product purified by column chromatography using silica and dichloromethane/*n*-pentane (3:2, R_f = 0.67) as eluent. The obtained solid was recrystallized from ethanol to give colourless needles (394 mg, 1.04 mmol, 77 %).

Melting point: 248 °C

^{1}H NMR (500MHz, $CDCl_3$, 300 K): δ = 4.44 (s, 4H), 0.30 (s, 18H) ppm;

^{13}C NMR (125 MHz, $CDCl_3$, 300 K): δ = 147.9 (2C), 117.4 (2C), 114.2 (2C), 112.7 (2C), 111.1 (2C), 94.7 (2C), 64.9 (2C), 0.3 (6C) ppm;

IR (ATR): ῦ = 2962 (w), 2899 (w), 2252 (w), 2168 (w), 1565 (m), 1462 (s), 1443 (s), 1375 (m), 1345 (s), 1309 (m), 1247 (s), 1127 (s), 1052 (m), 967 (s), 909 (s), 840 (vs), 759 (s), 703 (m), 663 (s) cm^{-1};

MS (EI, 70 eV): m/z (%) = 378 (18) $[M]^+$, 363 (100) $[M-Me]^+$;

HR-MS (EI, 70 eV): m/z = calcd. for $C_{20}H_{22}N_2O_2Si_2$ $[M]^+$ 378.1210, found 378.1213.

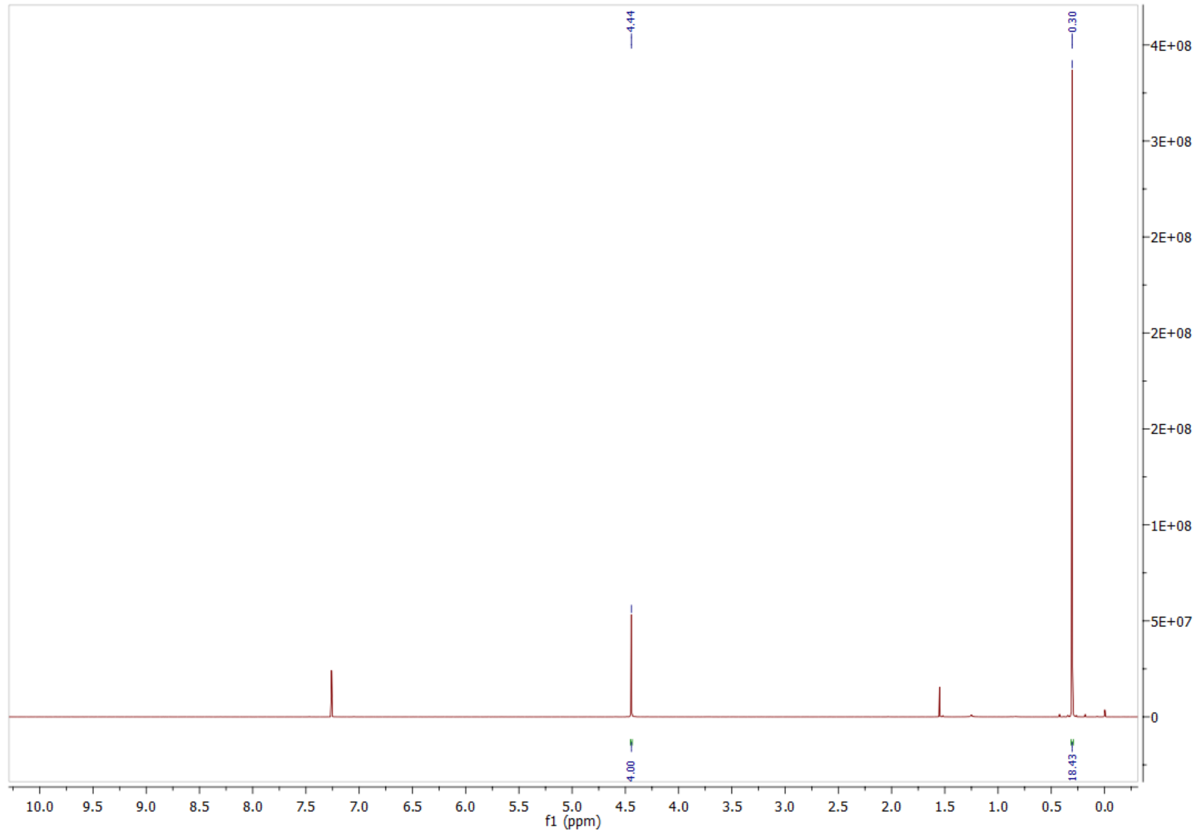

Figure S25: ^{1}H NMR spectrum of compound **16b** measured in deuterated chloroform.

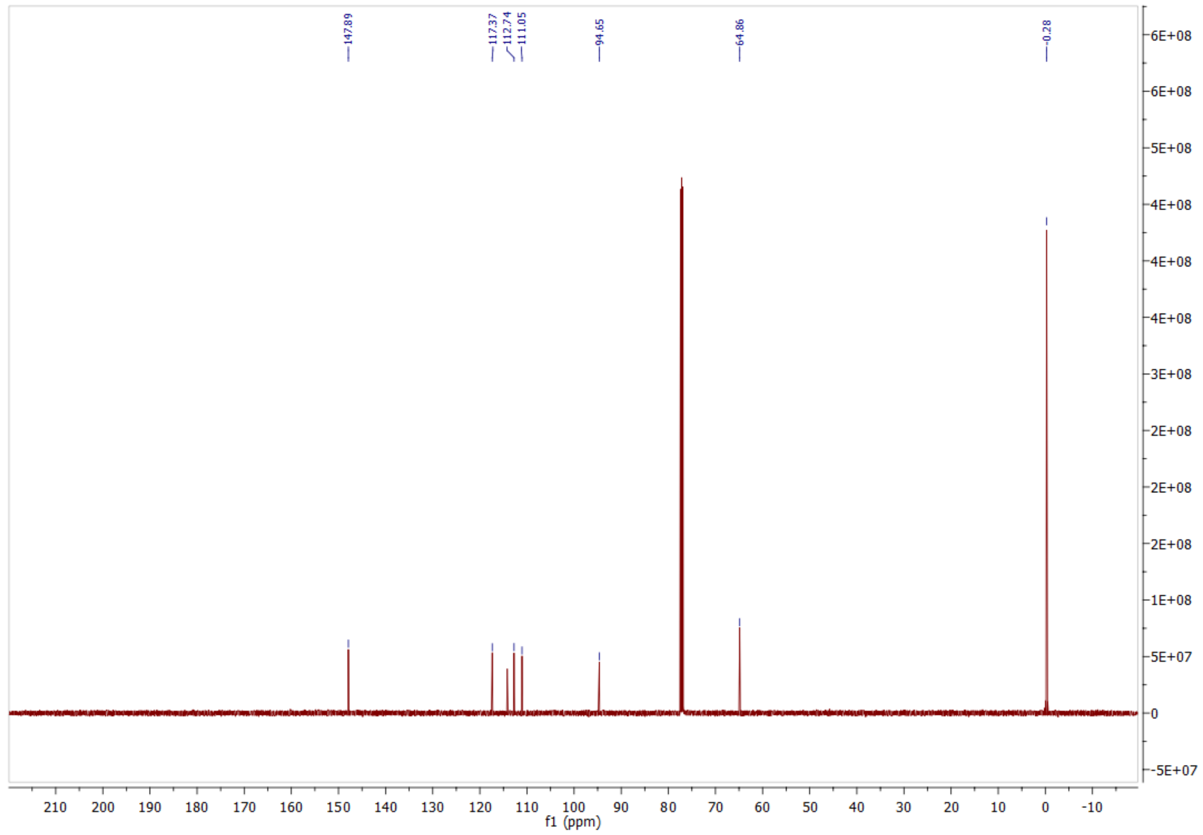

Figure S26: ^{13}C NMR spectrum of compound **16b** measured in deuterated chloroform.

3,3'-(6,7-Dicyano-1,4-benzodioxane-5,8-diyl)dipropiolic acid (5)

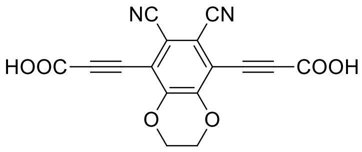

5

Under carbon dioxide atmosphere, 5,8-bis-(2-trimethylsilylethinyl)-6,7-dicyano-1,4-benzodioxane (**16b**, 278 mg, 734 µmol) was dissolved in anhydrous DMSO (4 mL), caesium fluoride (279 mg, 1.84 mmol) was added and the mixture stirred vigorously at room temperature for 18 h. Subsequently, the reaction mixture was diluted with water (400 mL), extracted with dichloromethane (3 × 150 mL) and the aqueous layer acidified with 6 M hydrogen chloride solution. The aqueous layer was extracted with diethyl ether (4 × 100 mL), the organic layer washed with 2 M hydrogen chloride solution (200 mL), dried over magnesium sulfate and the solvents removed in vacuo to obtain a pale brownish solid (191 mg, 593 µmol, 81%).

Melting point: >200 °C (slowly decomposing)

^{1}**H NMR** (500MHz, Acetone-d6, 300 K): δ = 4.70 (s, 4H), ppm;

^{13}**C NMR** (125 MHz, Acetone-d6, 300 K): δ = 153.4 (2C), 151.4 (2C), 115.2 (2C), 114.4 (2C), 113.2 (2C), 92.6 (2C), 75.5 (2C), 66.4 (2C) ppm;

IR (ATR): ῦ = 2900 (m_{br}), 2544 (m_{br}), 2226 (s), 1698 (vs), 1570 (s), 1467 (vs), 1449 (vs), 1387 (s), 1363 (s), 1301 (s), 1244 (vs), 1230 (vs), 1144 (s), 1056 (vs), 1019 (s), 989 (s), 937 (s), 834 (s), 792 (s), 717 (s), 748 (s), 675 (m) cm^{-1};

MS (EI, 70 eV): m/z (%) = 234 (100) $[M-2CO_2]^+$.

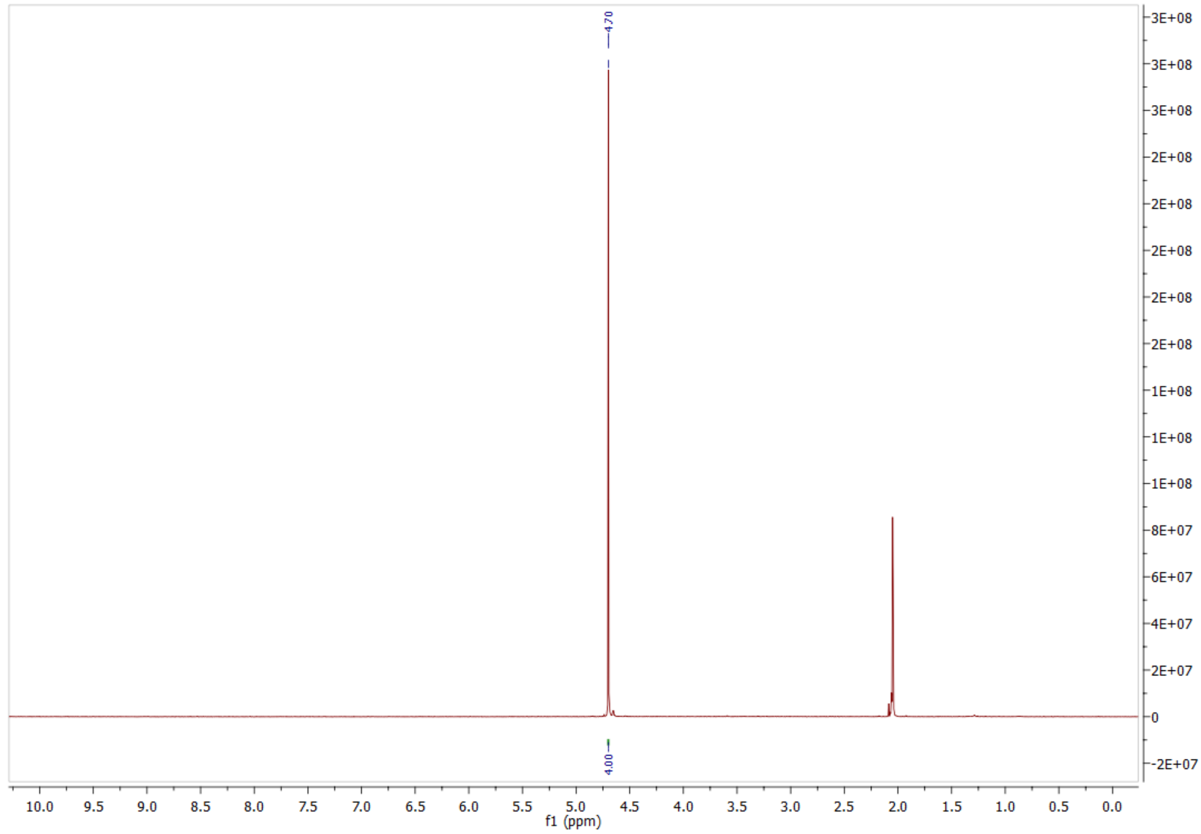

Figure S27: ^{1}H NMR spectrum of compound **5** measured in deuterated acetone.

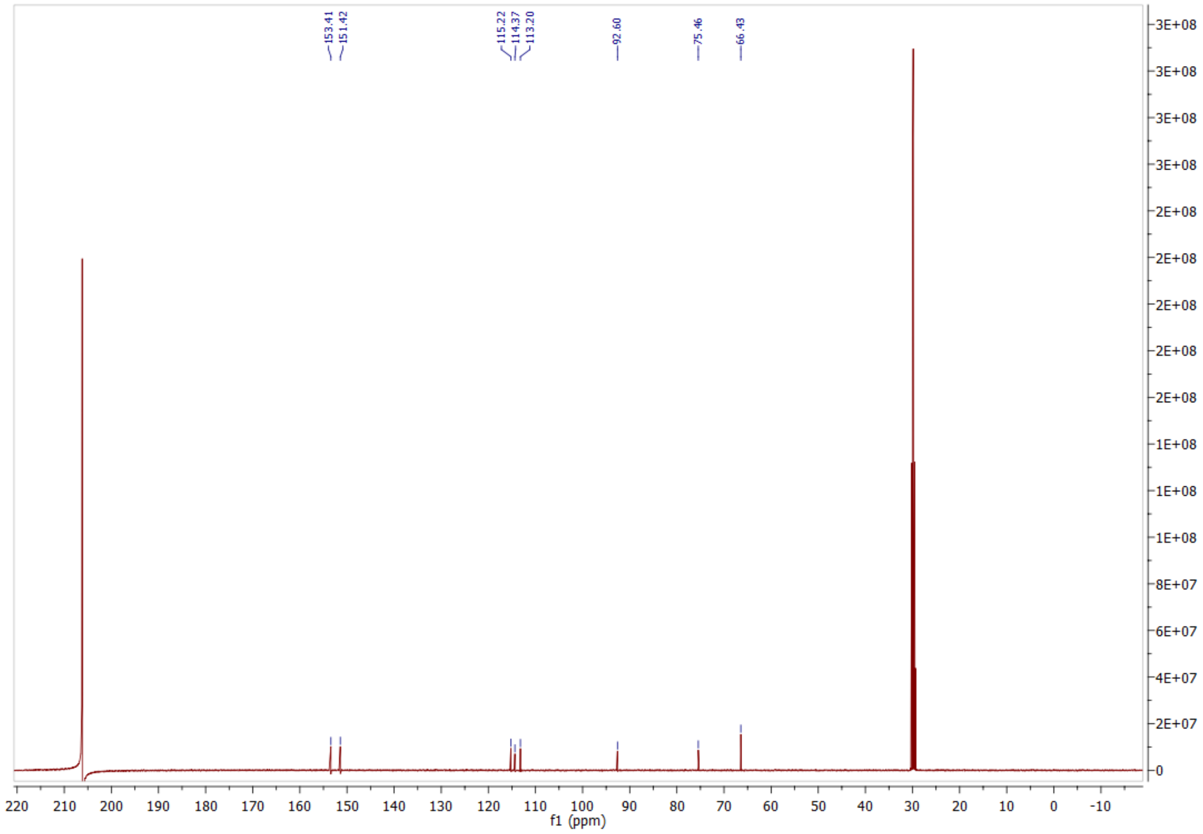

Figure S28: ^{1}H NMR spectrum of compound **5** measured in deuterated acetone.

III. UV–vis and fluorescence spectra

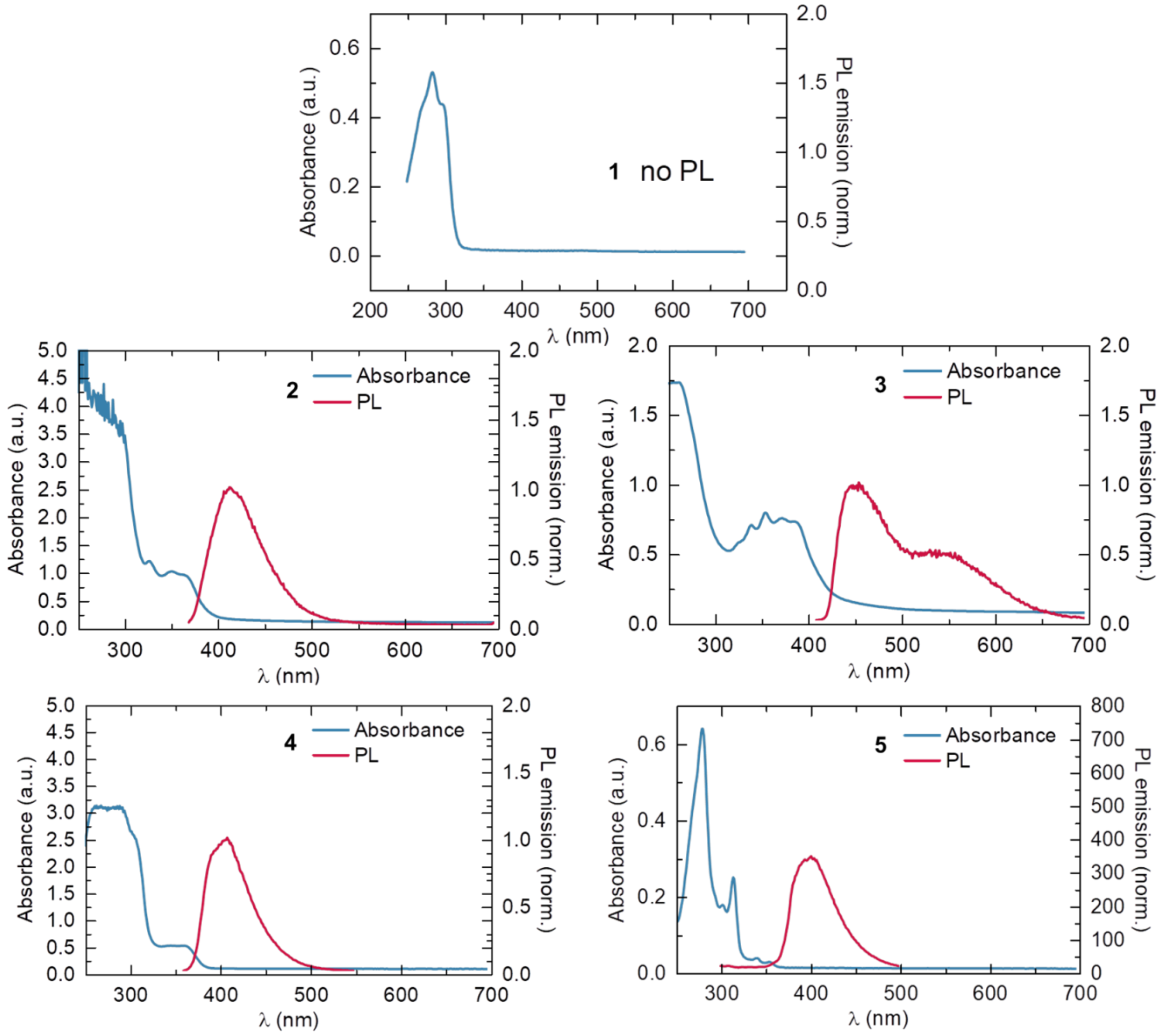

Figure S29: UV-Vis spectra of dipolar rotor compounds 1-5 (blue) as well as photoluminescence (red). Measurements were performed in ethanol solutions (40µM).

IV. Crystallographic data for compound 12b

Table S1. Crystal data and structure refinement for herges129.

Identification code	herges129	
Empirical formula	$C_{30}H_{42}N_4SSi_2$	
Formula weight	546.91	
Temperature	200(2) K	
Wavelength	0.71073 Å	
Crystal system	Monoclinic	
Space group	$P2_1/c$	
Unit cell dimensions	a = 13.3629(4) Å	α= 90°.
	b = 15.4255(3) Å	β= 94.777(2)°.
	c = 16.2296(4) Å	γ = 90°.
Volume	3333.78(14) $Å^3$	
Z	4	
Density (calculated)	1.090 Mg/m^3	
Absorption coefficient	0.192 mm^{-1}	
F(000)	1176	
Crystal size	0.06 × 0.12 × 0.18 mm^3	
Theta range for data collection	1.529 to 26.002°.	
Index ranges	-16<=h<=16, -19<=k<=16, -20<=l<=20	
Reflections collected	32011	
Independent reflections	6482 [R(int) = 0.0405]	
Completeness to theta = 25.242°	98.9 %	
Refinement method	Full-matrix least-squares on F^2	
Data / restraints / parameters	6482 / 0 / 346	
Goodness-of-fit on F^2	1.051	
Final R indices [I>2sigma(I)]	R1 = 0.0599, wR2 = 0.1623	
R indices (all data)	R1 = 0.0662, wR2 = 0.1677	
Extinction coefficient	n/a	
Largest diff. peak and hole	0.585 and -0.423 e.$Å^{-3}$	

Comments

Data collection was performed using an IPDS-2 from STOE & CIE. A numerical absorption correction was performed (Tmin(max: 0.9228/0.9828). The structure was solved with SHELXT and structure refinement was done with SHELXL-2014. All non-hydrogen atoms were refined anisotropic. The C-H H atoms were positioned with idealized geometry (methyl H atoms allowed to rotate but not to tip) and refined isotropic with $U_{iso}(H)$ = 1.2 $U_{eq}(C)$ (1.5 for methyl H atoms) using a riding model. Some of the isopropyl C atoms exhibits enlarged anisotropic displacement factors indicative for some static or dynamic disorder, which cannot be resolved.

CCDC-1896031contain the supplementary crystallographic data for this paper. These data can be obtained free charge from the Cambridge Crystallographic Data Centre via http://www.ccdc.cam.ac.uk/data_request/cif.

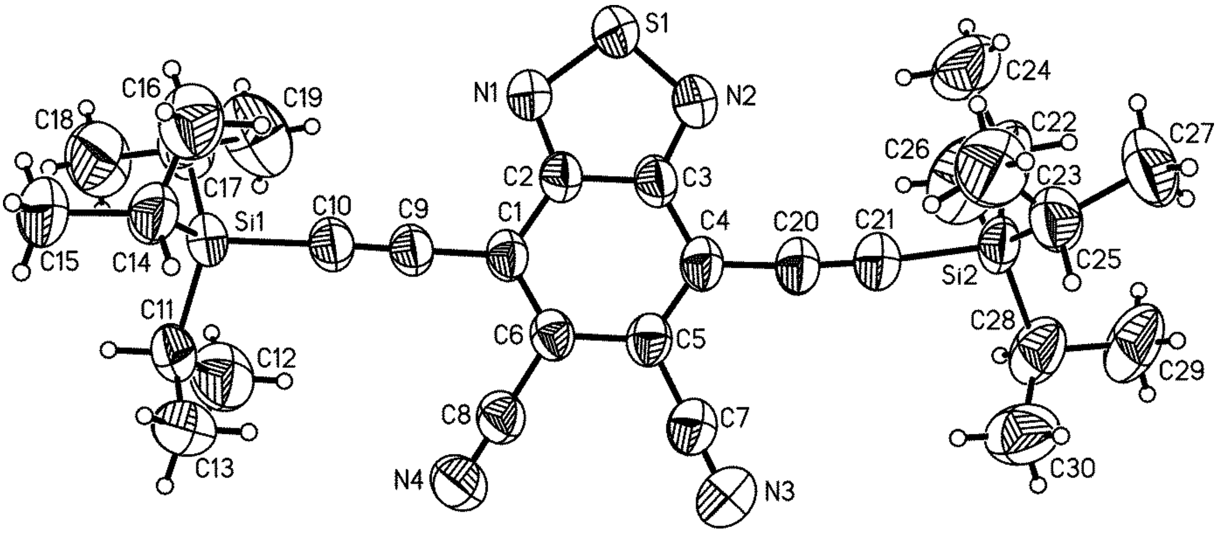

Figure S30: Crystal structure of **12b** with labeling and displacement ellipsoids drawn at the 50% probability level.

Table S2. Bond lengths [Å] and angles [°].

C(1)-C(6)	1.372(3)	S(1)-N(2)	1.597(2)	C(17)-C(18)	1.538(4)
C(1)-C(9)	1.427(3)	C(7)-N(3)	1.143(3)	C(20)-C(21)	1.190(3)
C(1)-C(2)	1.427(3)	C(8)-N(4)	1.141(3)	C(21)-Si(2)	1.841(2)
C(2)-N(1)	1.333(3)	C(9)-C(10)	1.190(3)	Si(2)-C(22)	1.866(3)
C(2)-C(3)	1.416(3)	C(10)-Si(1)	1.849(2)	Si(2)-C(25)	1.879(3)
C(3)-N(2)	1.343(3)	Si(1)-C(17)	1.874(3)	Si(2)-C(28)	1.895(3)
C(3)-C(4)	1.420(3)	Si(1)-C(11)	1.878(3)	C(22)-C(24)	1.509(5)
C(4)-C(5)	1.381(3)	Si(1)-C(14)	1.886(3)	C(22)-C(23)	1.514(6)
C(4)-C(20)	1.423(3)	C(11)-C(12)	1.515(5)	C(25)-C(26)	1.513(5)
C(5)-C(7)	1.431(3)	C(11)-C(13)	1.523(5)	C(25)-C(27)	1.530(4)
C(5)-C(6)	1.434(3)	C(14)-C(16)	1.519(4)	C(28)-C(30)	1.474(5)
C(6)-C(8)	1.437(3)	C(14)-C(15)	1.545(4)	C(28)-C(29)	1.546(4)
N(1)-S(1)	1.6127(19)	C(17)-C(19)	1.497(5)		
C(6)-C(1)-C(9)	122.81(19)	N(2)-S(1)-N(1)	101.18(10)	C(19)-C(17)-Si(1)	115.3(3)
C(6)-C(1)-C(2)	116.48(17)	C(3)-N(2)-S(1)	106.18(15)	C(18)-C(17)-Si(1)	112.1(2)
C(9)-C(1)-C(2)	120.69(19)	N(3)-C(7)-C(5)	179.2(3)	C(21)-C(20)-C(4)	175.1(3)
N(1)-C(2)-C(3)	113.70(19)	N(4)-C(8)-C(6)	178.5(2)	C(20)-C(21)-Si(2)	175.5(2)
N(1)-C(2)-C(1)	125.05(19)	C(10)-C(9)-C(1)	178.8(2)	C(21)-Si(2)-C(22)	105.65(12)
C(3)-C(2)-C(1)	121.2(2)	C(9)-C(10)-Si(1)	178.1(2)	C(21)-Si(2)-C(25)	106.13(12)
N(2)-C(3)-C(2)	113.1(2)	C(10)-Si(1)-C(17)	106.98(12)	C(22)-Si(2)-C(25)	114.63(17)
N(2)-C(3)-C(4)	125.48(19)	C(10)-Si(1)-C(11)	106.08(11)	C(21)-Si(2)-C(28)	105.41(13)
C(2)-C(3)-C(4)	121.40(19)	C(17)-Si(1)-C(11)	114.80(15)	C(22)-Si(2)-C(28)	109.35(16)
C(5)-C(4)-C(3)	116.73(18)	C(10)-Si(1)-C(14)	106.01(12)	C(25)-Si(2)-C(28)	114.78(15)
C(5)-C(4)-C(20)	123.4(2)	C(17)-Si(1)-C(14)	111.05(14)	C(24)-C(22)-C(23)	110.5(3)
C(3)-C(4)-C(20)	119.9(2)	C(11)-Si(1)-C(14)	111.31(13)	C(24)-C(22)-Si(2)	113.3(3)
C(4)-C(5)-C(7)	119.36(19)	C(12)-C(11)-C(13)	110.2(3)	C(23)-C(22)-Si(2)	112.2(3)
C(4)-C(5)-C(6)	121.66(19)	C(12)-C(11)-Si(1)	113.5(2)	C(26)-C(25)-C(27)	110.6(3)
C(7)-C(5)-C(6)	118.97(19)	C(13)-C(11)-Si(1)	111.8(2)	C(26)-C(25)-Si(2)	114.4(2)
C(1)-C(6)-C(5)	122.49(18)	C(16)-C(14)-C(15)	111.2(3)	C(27)-C(25)-Si(2)	112.5(3)
C(1)-C(6)-C(8)	118.89(18)	C(16)-C(14)-Si(1)	112.91(19)	C(30)-C(28)-C(29)	112.4(3)
C(5)-C(6)-C(8)	118.59(19)	C(15)-C(14)-Si(1)	112.0(2)	C(30)-C(28)-Si(2)	113.9(2)
C(2)-N(1)-S(1)	105.80(15)	C(19)-C(17)-C(18)	109.8(3)	C(29)-C(28)-Si(2)	111.7(3)

References

1. Zhang, Y.; Chien, S.-C.; Chen, K.-S.; Yip, H.-L.; Sun, Y.; Davies, J. A.; Chen, F.-C.; Jen, A. K.-Y. *Chem. Commun.*, **2011**, *47*, 11026–11028.

2. Chen, Z.; Cai, P.; Chen, J.; Liu, X.; Zhang, L.; Lan, L.; Peng, J.; Ma, Y.; Cao, Y. *Adv. Mater.*, **2014**, *26*, 2586–2591.

3. Jeong, I.; Chae, S.; Yi, A.; Kim, J.; Chun, H. H.; Cho, J. H.; Kim, H. J.; Suh, H. *Polymer*, **2017**, *109*, 115-125.

4. Viswanathan, V. N.; Rao, A. D., Pandey, U. K., Kesavan, A. V., Ramamurthy, P. C. *Beilstein J. Org. Chem.* **2017,** *13,* 863–873.

5. Wudarczyk, J.; Papamokos, G.; Marszalek, T.; Nevolianis, T.; Schollmeyer, D.; Pisula, W.; Floudas, G.; Baumgarten, M.; Müllen, K. *Appl. Mater. Interfaces*, **2017**, *9*, 20527−20535.

6. Shi, Q.; Zhang, S.; Zhang, J.; Oswald, V. F.; Amassian, A.; Marder, S. R.; Blakey, S. B. *J. Am. Chem. Soc.*, **2016**, *138*, 3946−3949.

7. Hellberg, J.; Dahlstedt, E.; Pelcman, M. E. *Tetrahedron*, **2004**, *60*, 8899–8912.

8. Daillaire, C.; Brook, M. A. *Organometallics*, **1993**, *12*, 2332-2338.

9. Fulmer, G. R.; Miller, A. J. M.; Sherden, N. H.; Gottlieb, H. E.; Nudelman, A.; Stoltz, B. M.; Bercaw, J. E.; Goldberg, K. I. *Organometallics*, **2010**, *29*, 2176-2179.
