# Supplementary material for: Synthesis of dipolar molecular rotors as linkers for metal-organic frameworks
Source: Beilstein J Org Chem. 2019 Jun 18;15:1331–8. doi: 10.3762/bjoc.15.132 (PMC6604738; doi:10.3762/bjoc.15.132)

BEILSTEIN JOURNAL OF ORGANIC CHEMISTRY

Supporting Information

for

Synthesis of dipolar molecular rotors as linkers for metal-organic frameworks

Sebastian Hamer, Fynn Röhricht, Marius Jakoby, Ian A. Howard, Xianghui Zhang, Christian Näther and Rainer Herges

Beilstein J. Org. Chem. **2019,** *15,* 1331–1338. doi:10.3762/bjoc.15.132

checkcif report for compound 12b

License and Terms: This is a supporting information file under the terms of the Creative Commons Attribution License (http://creativecommons.org/licenses/by/4.0). Please note that the reuse, redistribution and reproduction in particular requires that the authors and source are credited. The license is subject to the *Beilstein Journal of Organic Chemistry* terms and conditions: (https://www.beilstein-journals.org/bjoc)

checkCIF/PLATON report

You have not supplied any structure factors. As a result the full set of tests cannot be run.

THIS REPORT IS FOR GUIDANCE ONLY. IF USED AS PART OF A REVIEW PROCEDURE FOR PUBLICATION, IT SHOULD NOT REPLACE THE EXPERTISE OF AN EXPERIENCED CRYSTALLOGRAPHIC REFEREE.

No syntax errors found.   CIF dictionary   Interpreting this report

Datablock: herges129

```
Bond precision:  C-C = 0.0041 A          Wavelength=0.71073

Cell:            a=13.3629(4)     b=15.4255(3)       c=16.2296(4)
                 alpha=90         beta=94.777(2)     gamma=90
Temperature:     200 K

                Calculated                 Reported
Volume          3333.78(14)                3333.78(14)
Space group     P 21/c                     P 21/c
Hall group      -P 2ybc                    -P 2ybc
Moiety formula  C30 H42 N4 S Si2           ?
Sum formula     C30 H42 N4 S Si2           C30 H42 N4 S Si2
Mr              546.92                     546.91
Dx,g cm-3       1.090                      1.090
Z               4                          4
Mu (mm-1)       0.192                      0.192
F000            1176.0                     1176.0
F000'           1177.44
h,k,lmax        16,19,20                   16,19,20
Nref            6546                       6482
Tmin,Tmax       0.973,0.989                0.923,0.983
Tmin'           0.966

Correction method= # Reported T Limits: Tmin=0.923 Tmax=0.983
AbsCorr = NUMERICAL

Data completeness= 0.990          Theta(max)= 26.002

R(reflections)= 0.0599( 5702)     wR2(reflections)= 0.1677( 6482)

S = 1.051                  Npar= 346
```

The following ALERTS were generated. Each ALERT has the format
test-name_ALERT_alert-type_alert-level.
Click on the hyperlinks for more details of the test.

Alert level C

```
PLAT230_ALERT_2_C Hirshfeld Test Diff for   Si2      --C28       .        6.0 s.u.
PLAT241_ALERT_2_C High   'MainMol' Ueq as Compared to Neighbors of         S1 Check
PLAT242_ALERT_2_C Low    'MainMol' Ueq as Compared to Neighbors of        Si2 Check
PLAT242_ALERT_2_C Low    'MainMol' Ueq as Compared to Neighbors of        C17 Check
PLAT242_ALERT_2_C Low    'MainMol' Ueq as Compared to Neighbors of        C22 Check
PLAT242_ALERT_2_C Low    'MainMol' Ueq as Compared to Neighbors of        C25 Check
PLAT340_ALERT_3_C Low Bond Precision on  C-C Bonds ...............    0.00408 Ang.
```

Alert level G

```
PLAT230_ALERT_2_G Hirshfeld Test Diff for   Si1      --C10       .        6.2 s.u.
PLAT230_ALERT_2_G Hirshfeld Test Diff for   Si2      --C21       .        8.1 s.u.
PLAT371_ALERT_2_G Long   C(sp2)-C(sp1) Bond  C1       - C9       .       1.43 Ang.
PLAT371_ALERT_2_G Long   C(sp2)-C(sp1) Bond  C4       - C20      .       1.42 Ang.
PLAT933_ALERT_2_G Number of OMIT Records in Embedded .res File ...          1 Note
```

```
   0 ALERT level A = Most likely a serious problem - resolve or explain
   0 ALERT level B = A potentially serious problem, consider carefully
   7 ALERT level C = Check. Ensure it is not caused by an omission or oversight
   5 ALERT level G = General information/check it is not something unexpected

   0 ALERT type 1 CIF construction/syntax error, inconsistent or missing data
  11 ALERT type 2 Indicator that the structure model may be wrong or deficient
   1 ALERT type 3 Indicator that the structure quality may be low
   0 ALERT type 4 Improvement, methodology, query or suggestion
   0 ALERT type 5 Informative message, check
```

It is advisable to attempt to resolve as many as possible of the alerts in all categories. Often the minor alerts point to easily fixed oversights, errors and omissions in your CIF or refinement strategy, so attention to these fine details can be worthwhile. In order to resolve some of the more serious problems it may be necessary to carry out additional measurements or structure refinements. However, the purpose of your study may justify the reported deviations and the more serious of these should normally be commented upon in the discussion or experimental section of a paper or in the "special_details" fields of the CIF. checkCIF was carefully designed to identify outliers and unusual parameters, but every test has its limitations and alerts that are not important in a particular case may appear. Conversely, the absence of alerts does not guarantee there are no aspects of the results needing attention. It is up to the individual to critically assess their own results and, if necessary, seek expert advice.

Publication of your CIF in IUCr journals

A basic structural check has been run on your CIF. These basic checks will be run on all CIFs submitted for publication in IUCr journals (*Acta Crystallographica*, *Journal of Applied Crystallography*, *Journal of Synchrotron Radiation*); however, if you intend to submit to *Acta Crystallographica Section C* or *E* or *IUCrData*, you should make sure that full publication checks are run on the final version of your CIF prior to submission.

Publication of your CIF in other journals

Please refer to the *Notes for Authors* of the relevant journal for any special instructions relating to CIF submission.

PLATON version of 23/04/2018; check.def file version of 23/04/2018

Datablock herges129 - ellipsoid plot

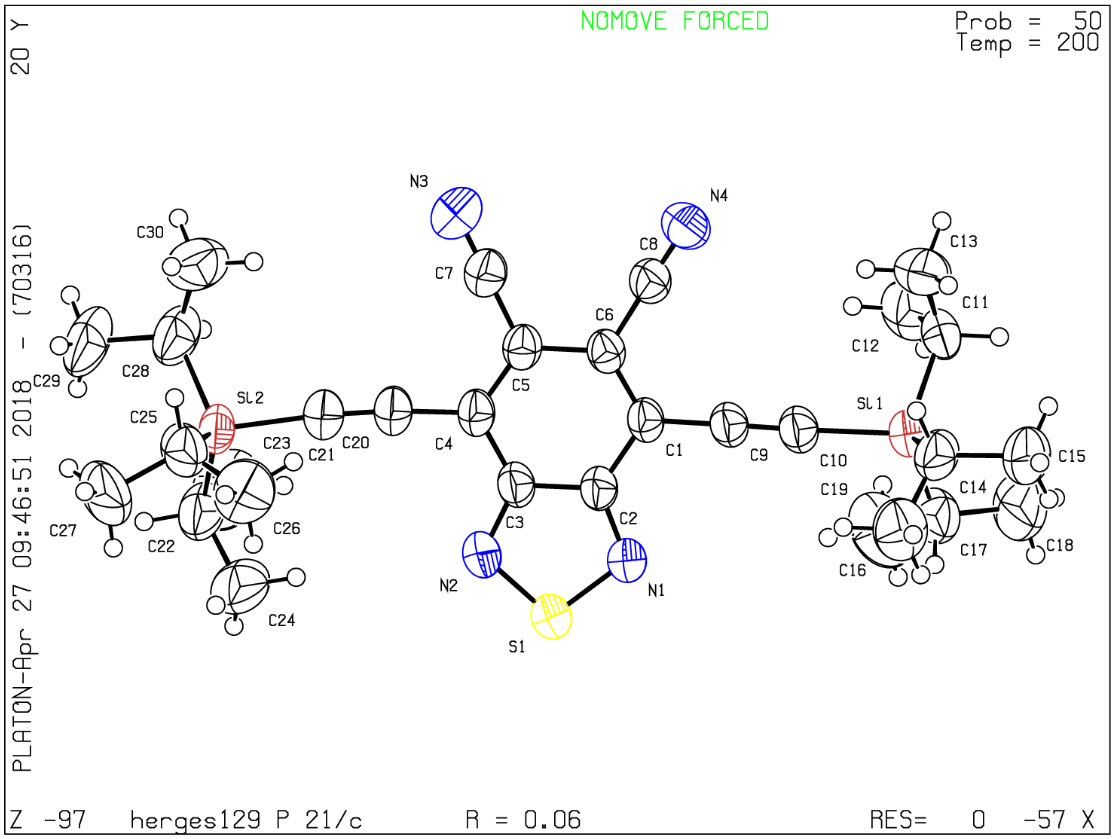

Supplement: File 3 — checkcif report for compound 12b. [file Beilstein_J_Org_Chem-15-1331-s003.pdf]
